# Ankyrin Is An Intracellular Tether for TMC Mechanotransduction Channels

**DOI:** 10.1016/j.neuron.2020.03.026

**Published:** 2020-07-08

**Authors:** Yi-Quan Tang, Sol Ah Lee, Mizanur Rahman, Siva A. Vanapalli, Hang Lu, William R. Schafer

**Affiliations:** 1 Neurobiology Division, MRC Laboratory of Molecular Biology, Francis Crick Avenue, Cambridge, UK; 2 School of Chemical and Biomolecular Engineering, Georgia Institute of Technology, Atlanta, GA 30332-0100, USA; 3 Department of Chemical Engineering, Texas Tech University, Lubbock, TX, USA; 4 Department of Biology, KU Leuven, 3000 Leuven, Belgium

**Keywords:** ankyrin, tether, CIB, TMC, mechanotransduction channel, gating spring, hair cells, C. elegans, nose touch

## Abstract

Mechanotransduction channels have been proposed as force sensors in various physiological processes, such as hearing and touch. In particular, TMC1 has been shown to constitute the pore of hair cell mechanotransduction channels, but little is known about how force is sensed by TMC channels. Here, we identify UNC-44/ankyrin as an essential component of the TMC-1 mechanotransduction channel complex in the sensory cilia of *Caenorhabditis elegans* mechanoreceptor neurons. Ankyrin binds indirectly to TMC-1 via evolutionarily conserved CIB proteins, which are required for TMC-1-mediated mechanosensation in *C. elegans* OLQ neurons and body wall muscles. Mechanosensory activity conferred by ectopically expressed TMCs in mechanoinsensitive neurons depends on both ankyrin and CIB proteins, indicating that the ankyrin-CIB subcomplex is required for TMC mechanosensitivity. Our work indicates that ankyrin is a long-sought intracellular tether that transmits force to TMC mechanotransduction channels.

## Highlights

• TMC-1 functions as a mechanosensor in *C. elegans* neurons and muscles
• UNC-44/ankyrin binds indirectly to TMC-1 via CALM-1
• CALM-1 and ankyrin are required for TMC-1-mediated mechanosensation
• Ankyrin acts as an intracellular tether to confer mechanosensitivity to TMC channels

## Graphical Abstract

**Figure:**
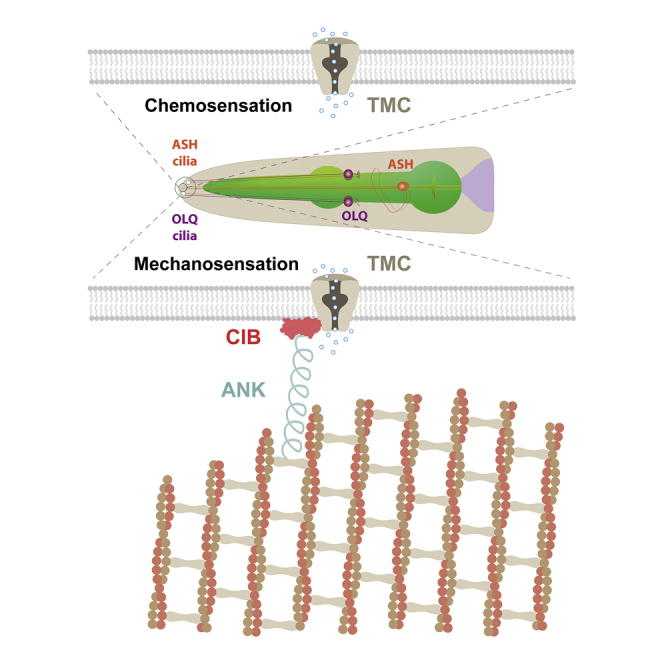

## Teaser

TMC channels are important for sensory transduction, notably as key components of the hair cell mechanotransducer. In this issue of *Neuron*, Tang et al. show that the mechanosensory activity of *C. elegans* TMCs requires the intracellular tether ankyrin, which interacts with TMC-1 through the adaptor protein CALM-1.

## Introduction

Our senses of touch, mechanical pain, hearing, balance, and proprioception all depend on mechanically activated ion channels. Two primary models have been put forward to understand how forces gate mechanotransduction channels (Ranade et al., 2015). The membrane force model supposes that the force is transmitted to the channel by membrane tension or bending of the lipid bilayer. Considerable evidence has been proposed to support membrane force model for bacterial MscS and MscL channels, eukaryotic potassium channels, and PIEZO channels (Brohawn et al., 2014, Ranade et al., 2015, Saotome et al., 2018, Zhao et al., 2018). In contrast, the tether model posits that the force is conveyed to open the channel by spring-like molecular tethers, either specialized domains of the channel subunit or accessory subunits, that bind to intracellular cytoskeletal elements and/or extracellular matrix (Ranade et al., 2015).

The tether model was first proposed for hair cells, the mechanosensory receptors of both auditory and vestibular systems in the vertebrate inner ear (Howard and Hudspeth, 1987, Howard and Hudspeth, 1988). Hair cells respond to the sound wave when this mechanical stimulus deflects hair bundles. Hair bundles consist of specialized microvilli called stereocilia, which are organized in three rows of increasing height and interconnected by tip links at their top. The transduction channels are localized at the tips of the shorter stereocilia and directly gated by mechanical forces imparted by hair bundle deflection (Beurg et al., 2009, Corey and Hudspeth, 1983). Biophysical characterization of hair bundle mechanics and channel gating suggests the existence of a gating spring, an elastic element that conveys force to the transduction channel. However, the molecular identity of the gating spring is controversial (Sotomayor et al., 2005). Although the tip link is thought to be an essential component of the mechanotransduction apparatus in hair cells (Assad et al., 1991), there remains a debate as to whether the gating spring is formed by the tip link itself or by unknown molecules connected in series with the tip link and/or intracellular cytoskeleton.

Current findings suggest that the mechanotransduction channel complex is composed of a number of proteins, including TMC1/2, LHFPL5, TMIE, and CIB2 (Giese et al., 2017, Pan et al., 2013, Pan et al., 2018, Xiong et al., 2012, Zhao et al., 2014). Recent evidence indicates that TMC proteins are pore-forming subunits of the hair cell mechanotransduction channel (Jia et al., 2020, Pan et al., 2018). However, it remains enigmatic how force is transmitted to TMC channels. In particular, TMC proteins also function as mechanosensors in *Drosophila* multidendritic neurons that lack complex hair-cell-specific structures, such as tip links (Guo et al., 2016, He et al., 2019, Zhang et al., 2016), suggesting that additional components may be required to convey forces to TMC channels.

In this study, we use genetically tractable model organism *C. elegans* to explore the mechanism for TMC mechanosensitivity. We show that worm TMC-1 is required for gentle nose-touch responses in mechanoreceptor OLQ neurons and body wall muscles. Yeast two-hybrid and proteomic screens identify CIB and ankyrin proteins as interacting components of the TMC-1 mechanotransduction channel complex, and these interactions appear to be essential for its function in touch sensing. Moreover, ectopic expression of *C. elegans* or human *TMC* genes in mechanoinsensitive ASK neurons generates ankyrin- and CIB-dependent mechanosensory activity, indicating that the ankyrin-CIB complex confers mechanosensitivity to TMC channels. These findings indicate that ankyrin acts as an intracellular tetherto confer force-sensing properties to TMC channels via CIB proteins, allowing them to function as mechanosensors.

## Results

### *C. elegans* TMC-1 Contributes to Mechanosensation in OLQ Neurons

**Figure 1:**
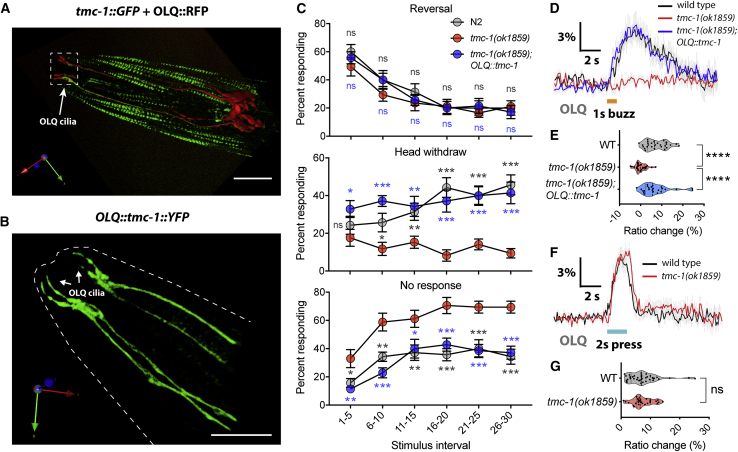
*C. elegans* TMC-1 Functions as a Mechanosensor in OLQ Neurons (A) 3D rendering reveals endogenous *tmc-1* expression in OLQ neurons, identified using *Pocr-4::RFP*. (B) 3D rendering reveals the OLQ ciliary localization of TMC-1 proteins. (C) Nose-touch behavior for wild type, *tmc-1(ok1859)* mutants, and *tmc-1(ok1859)* OLQ-rescued worms. n = 14–17. (D and F) Average traces of OLQ calcium responses to 1-s gentle (2.5 μm displacement) buzz (D) or 2-s harsh (8 μm displacement) press (F) stimulation in wild type, *tmc-1(ok1859)* mutants, and *tmc-1(ok1859)* OLQ-rescued worms. Gray shadings represent SEMs. The duration of the stimulus is shown in orange (D) or cyan (F). (E and G) Violin plots of OLQ calcium responses for all genotypes in (D) (E; n = 24–31) and (F) (G; n = 28–40). For (A) and (B), scale bars represent 10 μm. For (C), error bars indicate SEMs. For (E) and (G), data are presented as median with 25^th^ and 75^th^ percentile, and individual data points are plotted as shown. ns, not significant; ^∗^p < 0.05; ^∗∗^p < 0.01; ^∗∗∗^p < 0.001; ^∗∗∗∗^p < 0.0001. Statistical analyses were performed using multiple t tests with false discovery rate at Q = 1% (C), one-way ANOVA with Dunnett’s test (E), or unpaired t test (G).

To investigate the role of *C. elegans* TMC proteins *in vivo*, we tagged endogenous *tmc-1* with GFP at the C terminus via CRISPR/Cas9 genome editing to assess its cellular and subcellular localization. Because single-copy *tmc-1::gfp* knockin produces weak fluorescence signal *in vivo*, only those cells expressing high level of *tmc-1* could be detected by confocal microscopy. We observed expression of TMC-1::GFP fusion proteins in a few head and tail neurons, as well as vulval muscles and body wall muscles (Figures 1A and S1A–S1C). We did not observe expression in many other neurons that express *tmc-1* transcriptional reporters and where (in some cases) TMC-1 protein has been shown to function (Chatzigeorgiou et al., 2013, Wang et al., 2016, Yue et al., 2018, Zhang et al., 2015a), presumably because the expression level of TMC-1 is too low for detection from a single-copy transgenic strain. Notably, TMC-1::GFP was found to be enriched in the sensory cilia of mechanoreceptor OLQ neurons (Figures 1A, 1B, and S1D), implying a potential role in mechanosensation.

OLQ neurons are touch neurons with cilia in the outer labial sensillum on the worm’s nose, and OLQ ablation affects head withdrawal responses in response to nose touch (Kaplan and Horvitz, 1993, Kindt et al., 2007). We examined the nose-touch behavior in *tmc-1* mutant animals to determine whether head-withdrawal behavior was abnormal. Consistent with previous reports (Kindt et al., 2007), we found that, in wild type, animals initially responded to nose touch principally by switching from forward to backward crawling (reversal), but after consecutive touches, head withdrawal became the increasingly prevalent response (Figure 1C). In contrast, in *tmc-1* mutants, the frequency of head withdrawals was dramatically reduced, although no difference was seen in the frequency of reversal responses (Figures 1C and S1E). The head withdrawal defective phenotype of *tmc-1* mutants was substantially rescued by cell-specific expression of TMC-1 in OLQ neurons under the control of the *ocr-4* promoter (Figure 1C), indicating that *tmc-1* functions in OLQ neurons to promote nose-touch-evoked head withdrawal. These results indicate that TMC-1 is required in OLQ neurons for nose-touch sensation. Interestingly, our results also indicated that *tmc-1* did not affect nose-touch-evoked reversals, a response principally dependent on the FLP and ASH neurons (Kaplan and Horvitz, 1993). This is consistent with previous studies from different groups indicating that, although TMC-1 is important in ASH neurons for salt and alkaline chemosensation (Chatzigeorgiou et al., 2013, Wang et al., 2016), it may not contribute to mechanosensation in ASH.

To further investigate how TMC-1 might affect mechanosensory responses, we used the genetically encoded calcium indicator YC3.60 to monitor neural activity in response to mechanical stimuli in OLQ neurons. Two different types of mechanical stimuli were applied to the nose of the worm: a 1-s, small-displacement gentle buzz stimulus or a 2-s high-displacement harsh press stimulus. We observed that *tmc-1* mutants failed to respond to a gentle buzz, although wild-type worms exhibited robust calcium transients (Figures 1D and 1E). This defect could be rescued by OLQ-specific expression of *tmc-1* (Figures 1D and 1E), demonstrating that TMC-1 functions cell autonomously in OLQ neurons. However, we observed no significant difference between *tmc-1* mutants and wild-type animals in response to a harsh press (Figures 1F and 1G); this indicates that TMC-1 is not required for harsh touch responses in OLQs and that loss of *tmc-1* does not non-specifically compromise neuronal excitability or viability. Together, these results suggest that TMC-1 is specifically required for gentle touch responses in OLQ neurons, consistent with a direct role in mechanosensation.

### Identification of CIB Proteins as TMCs Binding Partners

**Figure 2:**
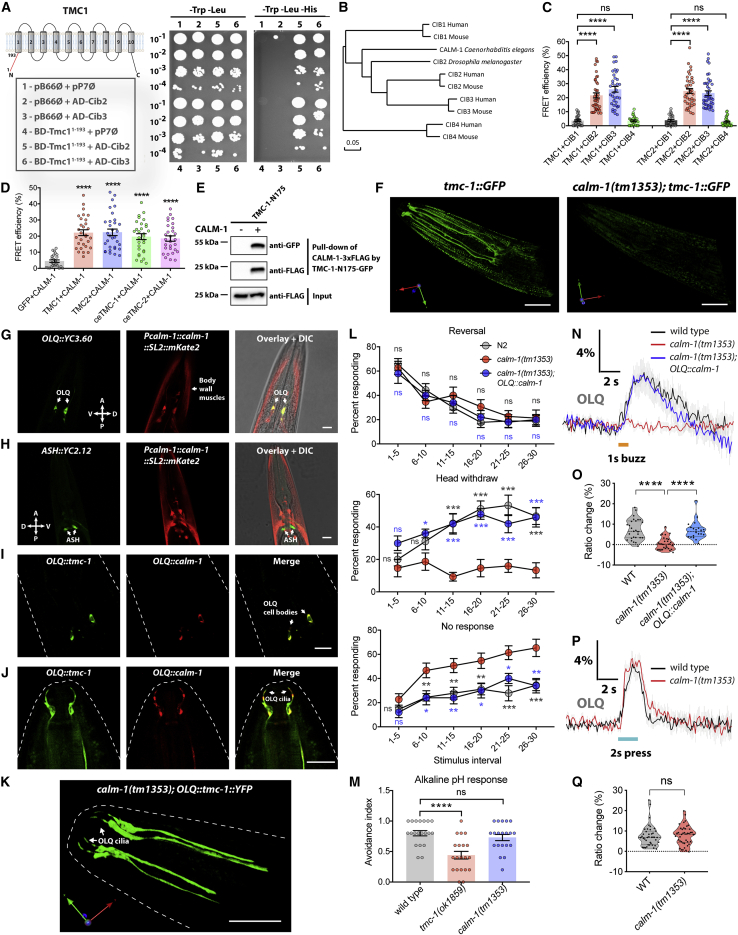
CALM-1 Is Required for TMC-1-Mediated Mechanosensation in OLQ Neurons (A) The cytoplasmic N terminus of mouse TMC1 (amino acid residues 1–193) interacts with CIB2 and CIB3 in yeast two-hybrid assays. Yeast cells carrying different combinations of constructs are listed on the left. Selective medium lacking tryptophan and leucine was used to control for growth and to verify the presence of both bait and prey plasmids (left). Protein-protein interactions were detected on a selective medium without tryptophan, leucine, and histidine (right; see STAR Methods). (B) The phylogenetic tree for *CIB* genes. A phylogenetic tree constructed by DNAMAN program illustrates the observed divergency of human, mouse, *Drosophila melanogaster*, and *Caenorhabditis elegans* CIB amino sequences, indicating that CIB2/3 are more closely related to *C. elegans* CALM-1 and that CIB1/4 are more evolutionarily distant. (C) Quantification of FRET efficiency indicates interactions between human TMC1/2 and CIB2/3, but not CIB1/4. n = 26–43. (D) Quantification of FRET efficiency indicates interactions of CALM-1 with *C. elegans* TMC-1/2 and human TMC1/2. n = 26–32. (E) Pull-down analysis of purified GFP-tagged TMC-1 N-terminal fragment (TMC-1-N175) and 3×FLAG-tagged CALM-1 proteins. Proteins are visualized by western blot. (F) 3D rendering of endogenous *tmc-1::GFP* expression in wild type and *calm-1* mutant. (G) Expression of *calm-1* in OLQ neurons, identified using *Pocr-4::YC3.60*. A SL2 *trans*-splicing sequence was used to separate the products of genes in operons, so the expression pattern of mKate2 can be used to indicate the expression pattern of *calm-1*. (H) Lack of expression of *calm-1* in ASH neurons, identified using *Psra-6::YC2.12*. (I and J) Co-localization of CALM-1 with TMC-1 in OLQ cell bodies (I) and cilia (J). (K) 3D rendering shows that the ciliary localization of TMC-1 in OLQ neurons is unaffected in *calm-1(tm1353)* mutants. (L) Nose-touch behavior for wild type, *calm-1(tm1353)* mutants, and *calm-1(tm1353)* OLQ-rescued worms. n = 10–18. (M) The *calm-1* mutant shows no defect in noxious alkaline-pH-induced avoidance behavioral response. n = 20. (N and P) Average traces of OLQ calcium responses to 1-s gentle buzz (N) or 2-s harsh press (P) stimulation in wild type, *calm-1(tm1353)* mutants, and *calm-1(tm1353)* OLQ-rescued worms. Gray shadings represent SEMs. The duration of the stimulus is shown in orange (N) or cyan (P). (O and Q) Violin plots of OLQ calcium responses for all genotypes in (N) (O; n = 20–29) and (P) (Q; n = 40–41). For (F)–(K), scale bars represent 10 μm. For (C), (D), (L), and (M), error bars indicate SEMs. For (O) and (Q), data are presented as median with 25^th^ and 75^th^ percentile, and individual data points are plotted as shown. ^∗^p < 0.05; ^∗∗^p < 0.01; ^∗∗∗^p < 0.001; ^∗∗∗∗^p < 0.0001. Statistical analyses were performed using one-way ANOVA with Dunnett’s test (C, D and M), multiple t tests with false discovery rate at Q = 1% (L), or unpaired t test (O and Q).

TMC-1 contributes to mechanosensation in OLQ neurons but only contributes to chemosensation in ASH neurons, implying that additional components, presumably intracellular tethers that transmit forces to the channel, may be required for it to function as a mechanosensory channel. We reasoned that such factors might be evolutionarily conserved proteins that are co-expressed with TMC proteins in mechanosensory cells, such as mammalian hair cells and worm OLQ neurons, but not in cells like ASH neurons. To identify such proteins, we carried out a yeast two-hybrid screening of a mouse inner ear cDNA library using the mouse TMC1 N terminus (amino acids 1–193) as bait. We chose the N terminus because it contains many charged amino acids and coiled-coil domains so that it could serve as a platform for protein-protein interactions. This screen identified two genes, *Cib2* and *Cib3*, both members of the calcium and integrin binding protein family (Figure 2A; Table S1). *CIB2* in humans is associated with nonsyndromic deafness (DFNB48; Riazuddin et al., 2012) and is essential for hearing and auditory hair cell mechanotransduction in mice (Giese et al., 2017, Michel et al., 2017, Wang et al., 2017). We then used the fluorescence resonance energy transfer (FRET) assay to investigate the interactions between TMC1/2 and CIB family members. In addition to *CIB2* and *CIB3*, vertebrate genomes contain two more evolutionarily divergent *CIB* genes, *CIB1* and *CIB4*, for which there is no ortholog in *C. elegans* (Figure 2B). We observed that both TMC1 and TMC2 interacted strongly with CIB2 and CIB3, but not with CIB1 and CIB4 (Figures 2C, S2A, and S2B), suggesting gene-specific interactions between CIB family and TMC family.

We next sought to identify key regions of CIB proteins required for the interaction with TMCs. Both human *CIB2* and *CIB3* generate multiple alternatively spliced transcripts (Figure S2C); in particular, long isoforms of *CIB2*/*3* (CIB2-1, CIB2-4, and CIB3-1) contain two exons encoding N-terminal regions of CIB proteins, which are missing in *CIB2/3* short isoforms (CIB2-2, CIB2-3, and CIB3-2). In the FRET assay, we observed strong associations between TMC1 and CIB2/3 long isoforms, but not the short isoforms (Figure S2C). Moreover, deletions of the N-terminal region of CIB2 abolished the associations with TMC1 (Figure S2D). In contrast, point mutations predicted to affect EF-hand calcium binding domains of CIB2 had no detectable effect on interactions with TMC1 (Figure S2E). Thus, TMC and CIB proteins appear to interact through their respective N-terminal domains.

### CIB2/3 Promotes TMC1/2 Protein Stability, but Not Cell-Surface Trafficking

The strong associations between TMC and CIB proteins raised the possibility that their interactions might be critical for either trafficking or function of TMC proteins. We therefore tested whether CIB2/3 promoted the exit of TMC1/2 from the endoplasmic reticulum (ER). Although CIB2/3 alone localized to the cytoplasm and cell periphery (Figure S2F), co-expression of full-length TMC1/2 resulted in the ER retention of the TMC1/2-CIB2/3 complex (Figures S2G and S2H), suggesting that CIB2/3 itself is not sufficient to promote trafficking of TMC1/2 to the cell surface, and CIB2/3 may assemble with TMC1/2 in the ER. However, CIB2/3 dramatically increased total TMC1/2 protein expression, whereas CIB1/4 had almost no effect (Figures S2I–S2L). Co-expression of either CIB2 or CIB3 significantly prolonged the half-life of TMC1/2 (Figures S2M–S2P), although the effect of CIB2 on TMC1 was less than that of CIB3, indicating that CIB2/3 increases TMC1/2 protein stability.

### CALM-1 Is Required for TMC-1-Mediated Mechanosensation in OLQ Neurons

*C. elegans* has a single gene ortholog to both *CIB2* and *CIB3*, called *calm-1* (Figure 2B). Human TMC1/2 and *C. elegans* TMC-1/2 all interacted strongly with CALM-1 (Figure 2D), and *C. elegans* TMC-1 also interacts with CALM-1 via its N terminus (amino acids 1–175) in the pull-down assay (Figure 2E), indicating that the mechanism of TMC-CIB interaction is evolutionarily conserved. To determine how CIB proteins affect TMC activity *in vivo*, we next explored the effect of *calm-1* mutationf on *tmc-1*-expressing cells. When we crossed our *tmc-1::gfp* knockin strain with the *calm-1*-null mutant, we observed that TMC-1::GFP protein expression was dramatically reduced (Figure 2F), consistent with our observations in mammalian cell lines (Figures S2I–S2L). These data suggest that CALM-1 boosts TMC-1 expression *in vivo*, making TMC-1::GFP readily detectable only in *calm-1*-expressing cells, such as OLQ neurons and body wall muscle cells, but not in *calm-1*- nonexpressing cells, such as ASH neurons (Figures 2G and 2H). We then examined whether CALM-1 associated and co-localized with TMC-1 *in vivo*. Remarkably, tagged CALM-1 and TMC-1 co-localized not only in the cell body but also in cilia of OLQ neurons (Figures 2I and 2J). Although the ciliary localization of TMC-1 in OLQ neurons is retained in *calm-1* mutant (Figure 2K), *calm-1* mutants showed similar behavioral defects as *tmc-1* mutants in nose-touch behavior (Figure 2L). In contrast, avoidance responses to alkaline pH were indistinguishable from wild-type animals, whereas *tmc-1* mutants were strongly defective (Figure 2M), indicating that CALM-1 is not required for TMC-1-mediated alkaline sensation in ASH neurons. Expression of a *calm-1* cDNA under the control of the *ocr-4* promoter rescued the head withdrawal defect of *calm-1* mutants (Figure 2L), indicating that *calm-1* functions in OLQ. We further measured OLQ neuronal activity in intact animals by calcium imaging and found that OLQ neurons of *calm-1* deletion mutants failed to exhibit calcium transients in response to gentle nose buzz stimuli (Figures 2N and 2O). This mechanosensitive response defect could be rescued by OLQ-specific expression of *calm-1* (Figures 2N and 2O). In contrast, the harsh press response in OLQ neurons, which does not require worm TMC-1, was not affected by *calm-1* deletion (Figures 2P and 2Q). These results suggest that CALM-1 co-localizes with TMC-1 in OLQ cilia, where mechanical forces are applied, and is specifically required for the mechanosensory role of TMC-1 in neurons.

### The TMC-1/CALM-1 Protein Complex Functions as a Mechanosensor in Body Wall Muscles of *C. elegans*

**Figure 3:**
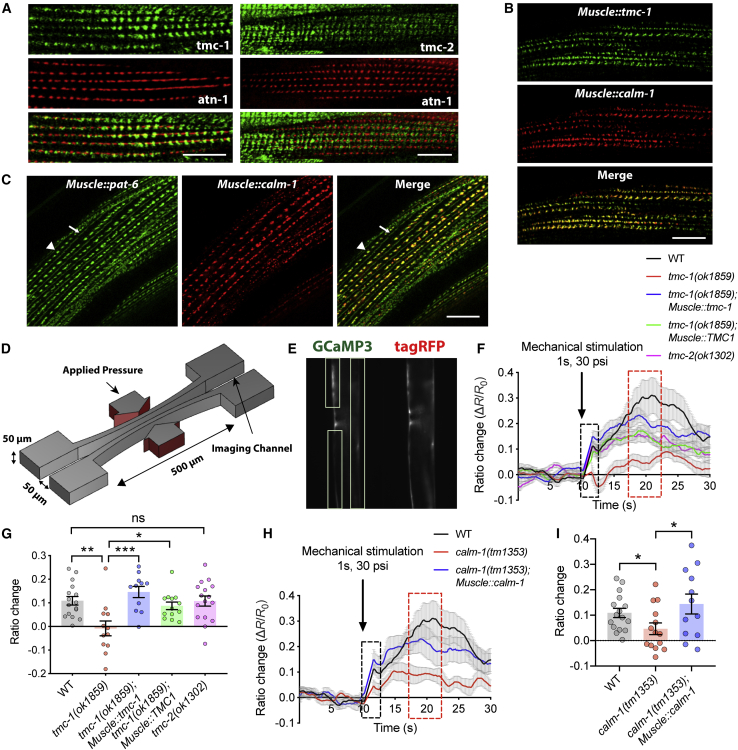
The TMC-1/CALM-1 Channel Complex Functions as a Mechanosensor in Body Wall Muscles of *C. elegans* (A) Left panel: localization of TMC-1::GFP at dense bodies as indicated by co-localization with mCherry::ATN-1. Right panel: localization of TMC-2::GFP near dense bodies as indicated by co-localization with mCherry::ATN-1 is shown. (B) Co-localization of CALM-1 with TMC-1 in body wall muscles. (C) Localization of CALM-1 to dense bodies as indicated by co-localization with PAT-6::GFP. Dense bodies (arrowheads) and M-lines (arrows) are indicated. (D) Schematic diagram of the PDMS actuator for the delivery of mechanical stimulation to worm body wall muscles. (E) Example images of a worm in the device for ratiometric calcium imaging. Dashed boxes indicate regions of interest for analysis. (F) Average traces of body wall muscle calcium responses to 1-s touch with 30 psi in wild type, *tmc-1(ok1859)* mutants, *tmc-1(ok1859)* muscle-rescued animals expressing *C. elegans* TMC-1 or human TMC1, and *tmc-2(ok1302)* mutants. (G) Quantification of initial calcium responses in body wall muscles for each genotype in (F). n = 11–16. (H) Average traces of body wall muscle calcium responses to 1-s touch with 30 psi in wild type, *calm-1(tm1353)* mutants, and *calm-1(tm1353)* muscle-rescued animals. (I) Quantification of initial calcium responses in body wall muscles for each genotype in (H). n = 12–16. For (A)–(C), scale bars represent 10 μm. For (F) and (H), gray shadings represent SEMs. Calcium traces in black dashed box indicate primary mechanosensory responses, and calcium traces in red dashed box indicate secondary responses. For (G) and (I), each data point indicates the average ratio change in a time window from 11.6 s to 12 s. Error bars indicate SEMs. ^∗^p < 0.05; ^∗∗^p < 0.01; ^∗∗∗^p < 0.001 (unpaired t test).

Given that both *tmc-1* and *calm-1* are expressed in body wall muscles of *C. elegans* (Figures 2F–2H), we wondered whether TMC-1 and CALM-1 might be required for mechanotransduction in muscles. Both TMC-1 and TMC-2 proteins are localized to the plasma membrane of body wall muscle cells, as indicated by the co-localization with a plasma membrane marker mCD8::mCherry (Figure S3A). However, only TMC-1 is localized to dense bodies where actin filaments are anchored and muscle contractile force is transduced to the plasma membrane (Figure 3A). Interestingly, CALM-1 is also co-localized with TMC-1 at dense bodies (Figures 3B and 3C). We therefore investigated whether the TMC-1/CALM-1 channel complex act as a mechanosensor at these contractile force transmission sites.

To test whether *C. elegans* muscle cells respond to mechanical stimuli, we used an automatic microfluidic device to deliver precise and repeatable mechanical stimuli to body wall muscles of *C. elegans* (Cho et al., 2017). Animals are loaded into an imaging channel that is well fitted to the worm’s body size so that they are trapped, but not physically restricted, in the imaging area, and mechanical stimuli are delivered through a pair of 500-μm-width actuated polydimethylsiloxane (PDMS) membranes (Figures 3D and 3E). In wild-type animals, we observed a quick and robust calcium response to a 1-s touch stimulus with 30 psi (Figures 3F and 3G; Video S1). This primary mechanical response was long-lasting; subsequently, a secondary calcium increase, probably caused by muscle contraction, was also observed (Figure 3F). When we applied touch stimuli to *tmc-1* mutants, such calcium responses were not observed (Figures 3F and 3G; Video S2). This defect could be rescued by muscle-specific expression of *tmc-1* (Figures 3F and 3G), demonstrating that TMC-1 functions cell autonomously in body wall muscles. Notably, human TMC1 could also rescue the mechanotransduction defect of *tmc-1* mutants, suggesting a functional conservation between human and nematode *TMC* genes. In contrast, we observed no significant difference between *tmc-2* mutants and wild-type animals in response to mechanical stimuli (Figures 3F and 3G). Given the different subcellular localization of TMC-1 and TMC-2 in body wall muscles (Figure 3A), TMC-1 and TMC-2 are unlikely to form heteromeric channels *in vivo*, and these two highly conserved channel subunits may play distinct roles in regulating muscle function. Similar to *tmc-1* mutants, *calm-1* mutants also showed dramatically reduced mechanically triggered activity (Figures 3H and 3I). This mechanosensitive response defect could be rescued by muscle-specific expression of *calm-1* (Figures 3H and 3I), suggesting that CALM-1 is cell autonomously required for TMC-1-mediated mechanosensation in body wall muscles.

Video S1. Mechanochip: Wild Type, Related to Figure 3 Shown is a wild-type animal carrying extrachromosomal array *ljIS131[Pmyo-3::GCaMP3-SL2-tagRFP-T]IV]* undergoing mechanical stimulation in the mechanochip.

Video S2. Mechanochip: Mutant, Related to Figure 3 Shown is a *tmc-1(ok1859)* animal carrying extrachromosomal array *ljIS131[Pmyo-3::GCaMP3-SL2-tagRFP-T]IV]* undergoing mechanical stimulation in the mechanochip.

### The TMC-1/CALM-1 Protein Complex Regulates Muscular Strength in *C. elegans*

Calcium signaling is critical for muscle function (Kuo and Ehrlich, 2015). To investigate whether TMC and CIB proteins could regulate *C. elegans* body wall muscle function, we utilized a recently developed microfluidics-based tool, NemaFlex (Rahman et al., 2018), to quantitatively analyze muscular strength of crawling *C. elegans*. The core of the NemaFlex technology is a liquid-filled microfluidic chamber consisting of an array of elastic PDMS micropillars dangling from the chamber roof that can be deformed by the push of a threading worm. Deflection measurements then allow us to calculate the force exerted by the worm via Timoshenko beam deflection theory for an elastic rod (Figure S3B). In *tmc-1* mutants, we observed a significant reduction in muscle strength (Figure S3C). In contrast, body wall muscles of *tmc-2* mutants, for which we had observed normal body muscle mechanosensory responses (Figures 3F and 3G), showed normal muscular strength (Figure S3C). The muscle weakness of *tmc-1* mutants and *tmc-1/tmc-2* double mutants could be rescued by muscle-specific expression of worm *tmc-1* or human *TMC1*, but not *tmc-2* (Figures S3D and S3E). *Calm-1* mutants also showed a defect in muscular strength (Figure S3F). Expression of a *calm-1* genomic DNA under its own promoter or a *calm-1* cDNA under *myo-3* muscle-specific promoter rescued the *calm-1* muscular strength defect (Figure S3F). These results collectively suggest that the TMC-1/CALM-1 protein complex regulates muscular strength in *C. elegans*.

### Identification of Ankyrin Proteins as Evolutionarily Conserved CIB-Interacting Proteins

**Figure 4:**
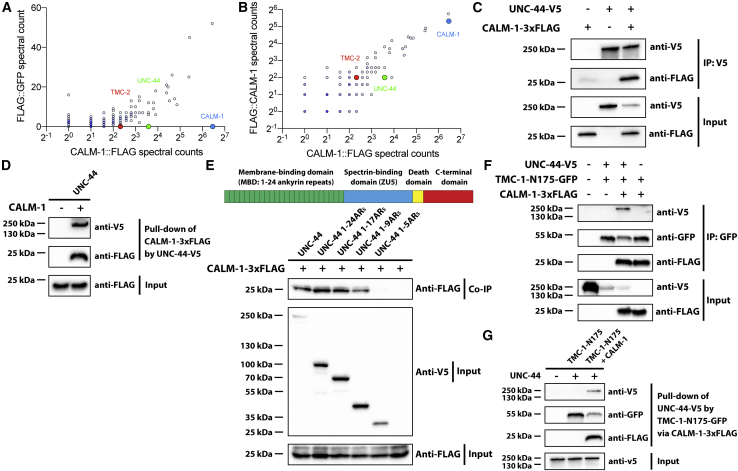
UNC-44/Ankyrin Binds Indirectly to TMC-1 via CALM-1 (A) Mass spectrometric analysis of proteins purified by anti-FLAG agarose beads from transgenic worms expressing *calm-1::FLAG* or *FLAG::gfp* under the control of the native *calm-1* promoter. The plot compares proteins co-precipitated with CALM-1::FLAG or the negative control FLAG::GFP. (B) Mass spectrometric analysis of proteins co-precipitated with CALM-1 tagged with FLAG at either the N or C terminus (FLAG::CALM-1/CALM-1::FLAG). (C) CoIP of CALM-1 with UNC-44 in HEK293T cells. (D) Pull-down analysis of purified V5-tagged UNC-44 and 3×FLAG-tagged CALM-1 proteins. Proteins are visualized by western blot. (E) Schematic diagram of UNC-44 (top) and coIP of CALM-1 with indicated deletion mutants of UNC-44 membrane-binding domain in HEK293T cells (bottom). (F and G) CoIP (F) and pull-down (G) analysis shows that the interaction between UNC-44 and TMC-1 N-terminal fragment (TMC-1-N175) requires CALM-1.

Although CALM-1 is crucial for TMC-1-mediated mechanosensation, it seems unlikely to act as an intracellular tether to transmit the force by itself. To understand the mechanism of how CALM-1 confers mechanosensitivity to TMC-1 channel, we thus performed a proteomic screen to search for CALM-1-interacting proteins (Figure S4A). Briefly, CALM-1 fused with FLAG at either the N or C terminus (FLAG::CALM-1/CALM-1::FLAG) or GFP fused with FLAG (FLAG::GFP) was transgenically expressed in worms using the native *calm-1* promoter. FLAG fusion proteins were purified from whole worm lysates using anti-FLAG antibody-conjugated magnetic beads, and mass spectrometry was performed to identify proteins that could be co-precipitated with both tagged forms of CALM-1, but not with FLAG-tagged GFP. One set of peptides corresponded to the known CALM-1-interacting protein TMC-2, validating our methodology. The absence of TMC-1 in our screen was presumably due to the low abundance of TMC-1 proteins in whole-worm lysates from our small-scale culture of worms. Among other hits, UNC-44, the sole ankyrin homolog in *C. elegans*, emerged as the strongest candidate protein in the screen (Figures 4A and 4B; Table S2).

Ankyrins are broadly expressed intracellular adaptors that link a variety of membrane protein complexes to the sub-membranous actin- and β-spectrin-based cytoskeleton (Bennett and Healy, 2008). Moreover, ankyrins contain 24 ankyrin repeats (AR_S_) that resemble a mechanosensitive helical spring (Jin et al., 2017, Lee et al., 2006, Zhang et al., 2015b), making them intriguing candidates for the elastic tether. To assess whether UNC-44 interacts with CALM-1 directly, we carried out coimmunoprecipitation (coIP), pull-down, and subcellular redistribution assays. UNC-44 coimmunoprecipitated with CALM-1 in extracts from HEK293T cells heterologously expressing V5-tagged UNC-44 (UNC-44-V5) and 3×FLAG-tagged CALM-1 (CALM-1-3×FLAG; Figure 4C). Similarly, CALM-1 could also be pulled down by anti-V5 agarose-beads-immobilized UNC-44-V5, suggesting a direct interaction between UNC-44 and CALM-1 (Figure 4D). In the third assay, the CAAX motif from K/H-Ras signaling protein was appended to the C terminus of CALM-1::3×FLAG fusion protein to function as a membrane targeting signal (MTS) that translocates the fusion protein to the inner leaflet of the plasma membrane (Figures S4B and S4C; Tang et al., 2013). Overexpressed exogenous CALM-1 with CAAX motifs (CALM-1-MTS), which is normally localized to the plasma membrane, is recruited to the ER when CALM-1 binding partner TMC-1 is co-expressed (Figure S4D). Co-expression of UNC-44 also resulted in efficient cytoplasmic recruitment of CALM-1-MTS (Figure S4E), but not GFP-MTS (Figure S4F), whereas non-interacting protein LHFPL5 failed to recruit CALM-1-MTS to the cytoplasm (Figure S4G).

Ankyrins interact with diverse membrane proteins via their N-terminal membrane-binding domains (MBDs). To assess the structural requirements on UNC-44 for direct CALM-1 binding, we used a series of UNC-44 MBD mutants. We found that, although full-length MBD (1–24AR_S_), 1–17AR_S_, and 1–9AR_S_ interacted with CALM-1, 1–5AR_S_ failed to bind to CALM-1 (Figure 4E), indicating that the structural integrity of AR_S_ is required for CALM-1 binding.

To test whether CALM-1 is necessary for the interaction between ankyrin and TMC proteins, we then evaluated direct protein-protein interaction between V5-tagged UNC-44 (UNC-44-V5) and GFP-tagged TMC-1 N-terminal fragment (TMC-1-N175-GFP) using coIP and pull-down assays. TMC-1-N175 only coimmunoprecipitated UNC-44 in the presence of co-expressed CALM-1 (Figure 4F). Similarly, GFP-Trap beads-immobilized TMC-1-N175-GFP only pulled down UNC-44 in the presence of CALM-1 (Figure 4G). These results suggest that there could be a large ankyrin-repeat structure, which provides enough extensibility and elasticity to serve as a gating spring, between the TMC-CIB channel complex and the intracellular cytoskeleton.

### UNC-44/Ankyrin Is Required for TMC-1-Mediated Mechanosensation in OLQ Neurons

**Figure 5:**
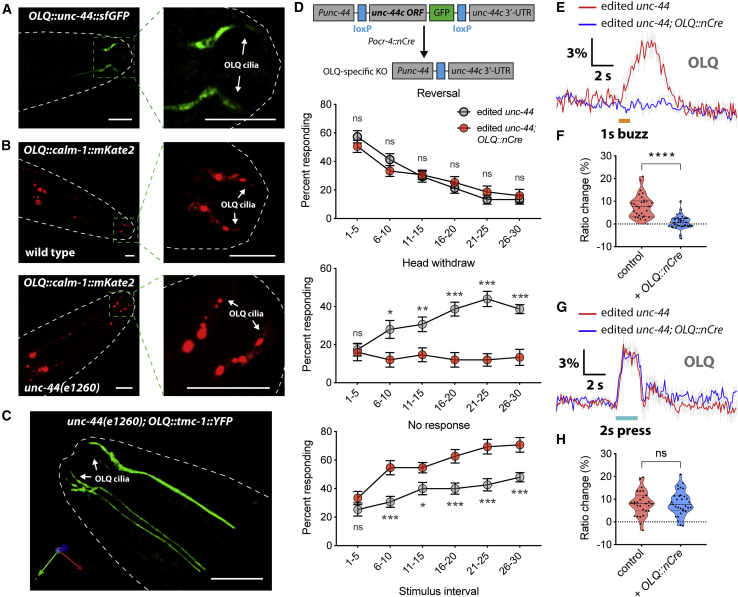
UNC-44/Ankyrin Is Required for TMC-1-Mediated Mechanosensation in OLQ Neurons (A) Ciliary localization of UNC-44 in OLQ neurons. (B) Ciliary localization of CALM-1 in wild-type and *unc-44*-deficient OLQ neurons. (C) 3D rendering of endogenous *tmc-1::GFP* expression in unc*-44* mutant. (D) Schematic of OLQ-specific *unc-44* knockout strategy (top) and nose-touch behavior for *unc-44*-edited and OLQ-specific *unc-44* knockout worms (bottom). Using CRISPR/Cas9, one loxP site was inserted just upstream of the *unc-44* start codon, and GFP and another loxP site were inserted after Leu1818 in an exon present in most *unc-44* isoforms. To achieve cell-type-specific knockout of *unc-44* in this edited strain, nuclear localized Cre recombinase (nCre) was expressed under cell-specific promoters, leading to excision of *unc-44* exons. n = 15. (E and G) Average traces of OLQ calcium responses to 1-s gentle buzz (E) or 2-s harsh press (G) stimulation in *unc-44*-edited and OLQ-specific *unc-44* knockout worms. Gray shadings represent SEMs. The duration of the stimulus is shown in orange (E) or cyan (G). (F and H) Violin plots of OLQ calcium responses for all genotypes in (E) (F; n = 29–36) and (G) (H; n = 29–32). For (A)–(C), scale bars represent 10 μm. For (D), error bars indicate SEMs. For (F) and (H), data are presented as median with 25^th^ and 75^th^ percentile, and individual data points are plotted as shown. ^∗^p < 0.05; ^∗∗^p < 0.01; ^∗∗∗^p < 0.001; ^∗∗∗∗^p < 0.0001. Statistical analyses were performed using multiple t tests with false discovery rate at Q = 1% (D) or unpaired t test (F and H).

We next sought to evaluate whether UNC-44/ankyrin is required for TMC-1-mediated mechanosensation in OLQ cilia *in vivo*. We first examined its role in localization of the TMC-1/CALM-1 mechanotransduction channel complex in OLQ neurons. UNC-44 is localized to the OLQ cilia, as are TMC-1 and CALM-1 (Figure 5A). OLQ cilia appear structurally normal in *unc-44* mutants (Figure S5A). Thus, UNC-44 does not play an essential role in specifying or maintaining OLQ cilium structure in *C. elegans*. Furthermore, TMC-1::GFP and CALM-1::mKate2 localization along the axoneme of OLQ cilia is retained in *unc-44* mutants (Figures 5B and 5C). These results suggest that UNC-44/ankyrin is not required for TMC-1 and CALM-1 ciliary localization in OLQ neurons.

To characterize the role of *unc-44* in nose-touch behavior, we used CRISPR/Cas9 editing to insert two loxP sites into the endogenous *unc-44* locus to permit cell-specific knockout of *unc-44* (Figure 5D). The knockin of loxP sites does not disrupt *unc-44* function, as the edited animals superficially resembled the wild type in locomotion and development (Video S3). Expression of a nuclear localized Cre recombinase (nCre) under cell-specific promoters cleaved the *unc-44* coding region in specific cell types. To validate this approach, we expressed nCre under a pan-neuronal promoter (*Prab-3::nCre*) in this edited *unc-44* strain; this resulted in locomotion defects reminiscent of *unc-44* mutants (Video S4). We next specifically deleted *unc-44* in OLQ neurons by expressing nCre under a OLQ neuron-specific promoter (*Pocr-4::nCre*) and examined the nose-touch behavior. Similar to deletion of *tmc-1* and *calm-1*, OLQ-specific knockout of *unc-44* led to reduced head withdrawal responses but had no effect on reversal responses to nose touch (Figure 5D). We further measured OLQ neuronal activity in OLQ-specific *unc-44* knockout animals by calcium imaging. CRISPR/Cas9-mediated GFP knockin did not interfere imaging of the YC3.60 calcium indicator, as the expression level of endogenous *unc-44* is much lower than overexpressed YC3.60 (Figure S5B). We observed that *unc-44*-deficient OLQ neurons failed to exhibit TMC-1-dependent calcium transients in response to gentle nose buzz stimuli (Figures 5E and 5F). In contrast, the harsh press response in OLQ neurons, which does not require TMC-1, was not affected by *unc-44* deletion (Figures 5G and 5H). These results illustrate that UNC-44/ankyrin acts cell autonomously as an essential component of the TMC-1 mechanotransduction channel complex in OLQ neurons.

Video S3. unc44cre: Control, Related to Figure 5 Shown is an animal with the *unc-44* genome editing cassette before recombination.

Video S4. unc44cre: Knockout, Related to Figure 5 Shown is an animal with the *unc-44* genome editing cassette after pan-neuronally expressed recombinase has generated a deletion mutation in neurons.

### Ectopic Expression of TMC Proteins Confers CIB/Ankyrin-Dependent Mechanosensitivity to *C. elegans* Chemosensory Neurons

**Figure 6:**
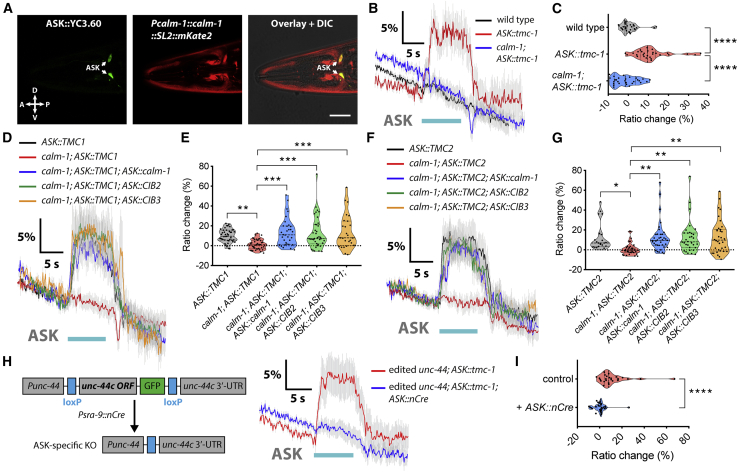
Ectopic Expression of TMCs Confers CIB/Ankyrin-Dependent Mechanosensitivity to ASK Neurons (A) Expression of *calm-1* in ASK neurons, identified using *Psra-9::YC3.60*. Scale bar represents 20 μm. (B, D, and F) Average calcium traces of wild-type ASK neurons or ASK neurons ectopically expressing worm *tmc-1* (B), human *TMC1* (D), and *TMC2* (F) in wild type, *calm-1(tm1353)* mutants, or *calm-1(tm1353)* ASK-rescued animals in response to 10-s press stimulation. (C, E, and G) Violin plots of ASK calcium responses for all genotypes in (B) (C; n = 28), (D) (E; n = 26–43), and (F) (G; n = 22–37). (H) Schematic of ASK-specific *unc-44* knockout strategy (left) and average calcium traces of ASK neurons ectopically expressing worm *tmc-1* in *unc-44*-edited and ASK-specific *unc-44* knockout animals in response to 10-s press stimulation (right). (I) Violin plots of ASK calcium responses for all genotypes in (H). n = 25–28. For (B), (D), (F), and (H), gray shadings represent SEMs. The duration of the stimulus is shown in cyan. For (C), (E), (G), and (I), data are presented as median with 25^th^ and 75^th^ percentile, and individual data points are plotted as shown. ^∗^p < 0.05; ^∗∗^p < 0.01; ^∗∗∗^p < 0.001; ^∗∗∗∗^p < 0.0001. Statistical analyses were performed using one-way ANOVA with Dunnett’s test (C, E, and G) or unpaired t test (I).

Given that both CALM-1 and UNC-44 are required for TMC-1-mediated mechanosensation in OLQ neurons, we therefore tested whether CALM-1 and UNC-44 could potentiate TMC-dependent mechanosensory activity in cells that do not endogenously express TMC proteins. Using a reporter transgene, we determined that *calm-1* is expressed in chemosensory ASK neurons (Figure 6A), which lack endogenous mechanosensory responses and do not express *tmc-1* and *tmc-2* (Figures S6A and S6B). We then tested whether expression of worm TMC-1 or mammalian TMC1/2 conferred ectopic mechanosensitivity to ASK neurons. We found that, in animals expressing either *tmc-1* or *TMC1/2* transgene, ASK neurons exhibited robust responses to both continuous pressure (“press”) and vibrational (“buzz”) stimuli applied near ASK cell body although wild-type ASK failed to respond to press (Figures 6B–6G and S6C–S6G). In contrast, heterologous ASK expression of two other components required for hair cell mechanotransduction (Xiong et al., 2012, Zhao et al., 2014), *Lhfpl5* and *Tmie*, did not lead to ectopic mechanical responses (Figures S6H and S6I). Deletion of *calm-1* eliminated TMC-dependent mechanosensory responses, which could be restored by cell-specific expression of CALM-1 or human CIB2/3 in ASK neurons (Figures 6D–6G). Similarly, cell-specific deletion of *unc-44* in ASK neurons also led to loss of mechanosensory activity of TMC-1 channels (Figures 6H and 6I). Therefore, TMC mechanosensitivity appears to be CIB and ankyrin dependent in *C. elegans* neurons.

### The CIB-Ankyrin Complex Is Directly Required for TMC Mechanosensitivity

**Figure 7:**
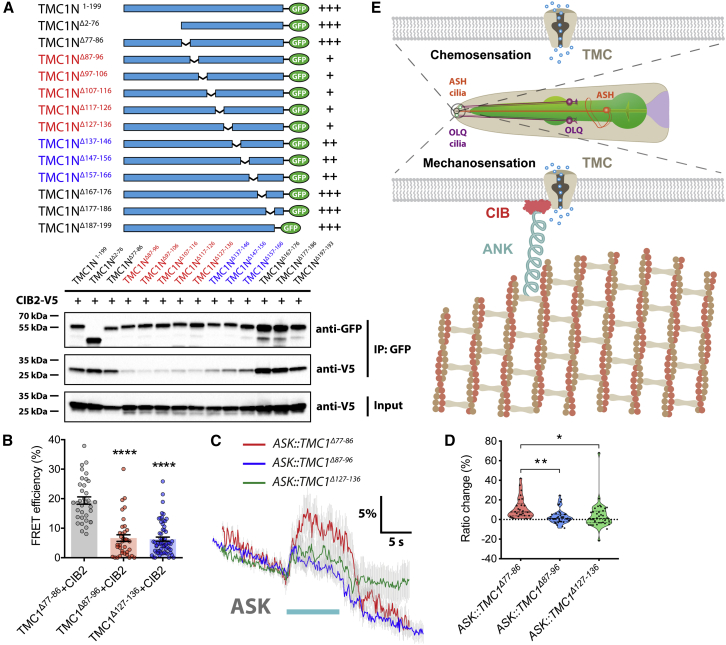
TMC Mechanosensitivity Depends on Binding of Ankyrin-CIB (A) Schematic of TMC1 N-terminal deletion constructs (top). Black thin lines indicate deleted regions, and cyan solid boxes represent cytoplasmic N-terminal coding regions. CoIP of CIB2 with indicated deletion mutants of TMC1 N-terminal regions is shown. Multiple interacting domains within TMC1 N terminus are required for CIB2 binding (bottom). (B) Quantification of FRET efficiency indicates that deletions of amino acid residues 87–96 and 127–136 disrupt the interaction between TMC1 and CIB2, whereas deletion of adjacent residues 77–86 does not. n = 35–62. (C) Average traces of calcium responses to 10-s press stimulation in ASK neurons ectopically expressing truncated *TMC1* mutants. Gray shadings represent SEMs. The duration of the stimulus is shown in cyan. (D) Violin plots of ASK calcium responses for all genotypes in (C). n = 26–37. (E) Model of TMC in mechanosensation and chemosensation. For (B), error bars indicate SEMs. For (D), data are presented as median with 25^th^ and 75^th^ percentile, and individual data points are plotted as shown. ^∗^p < 0.05; ^∗∗^p < 0.01; ^∗∗∗∗^p < 0.0001. Statistical analyses were performed using one-way ANOVA with Dunnett’s test.

Loss of CIB and UNC-44 expression might affect TMC channels indirectly through regulating other molecules. To investigate whether the ankyrin-CIB complex is directly required for TMC mechanosensitivity, we generated deletion mutations affecting the TMC1 N terminus and assessed their impact on interactions with CIB proteins and on heterologous touch responses in ASK neurons. We found that deleting residues 87–136 in TMC1 significantly disrupted the interaction between TMC1 N terminus and CIB2 and deleting residues 137–166 moderately disrupted the interaction (Figure 7A) when assayed by coIP experiments, suggesting that multiple interaction domains may exist within the TMC1 N terminus. Although the amino acid sequences of TMC N termini are variable, the regions (from amino acid 87 to 166 in human TMC1) required for interactions with CIB2 are well conserved and similar across different TMC family members and species (Figures S7A and S7B). CoIP and FRET assays further confirmed that residues 87–96 and 127–136 were critical for associations of TMC1 protein with both CIB2 and CIB3 (Figures 7B and S7C–S7E). When we expressed these mutant forms of *TMC1* (Δ87–96 and Δ127–136) in ASK neurons, we observed significantly reduced responses to mechanical stimuli, whereas a deletion mutant affecting an immediately adjacent region (Δ77–86) that did not disrupt interactions with CIB2/3 showed similar responses to wild-type *TMC1* (Figures 7C and 7D). Together, these results further suggest that direct interactions between CIB proteins and ankyrins, and between CIB proteins and the TMC pore-forming subunits, are required to mechanically gate the channel (Figure 7E).

## Discussion

In this study, we have identified CIB and ankyrin proteins as evolutionarily conserved components of the TMC mechanotransduction channel complex and provided evidence that the ankyrin-CIB complex is essential for TMC mechanosensory gating. We further show that heterologous expression of TMCs can render chemosensory neurons mechanosensitive in a CIB/ankyrin-dependent manner, highlighting the functional significance of ankyrin-CIB for mechanosensation. Our study supports an intracellular tether mechanism for TMC mechanosensitivity, providing fundamental insights into the molecular basis of mechanotransduction.

### Mechanosensitivity of TMC Channels

TMC1 has been proposed to be the pore-forming subunit of the hair cell mechanotransduction channel (Pan et al., 2018). However, whether TMC channels are inherently mechanosensitive or whether they require auxiliary subunits to sense forces remains unknown. Here, we show that TMC-1 contributes to mechanosensory responses in *calm-1/unc-44*-expressing OLQ nose-touch neurons. In contrast, TMC-1 is important for chemosensation, but not nose touch sensation in ASH neurons, suggesting that TMC-1 alone does not contribute to mechanosensation in neurons that do not express the ankyrin-CIB complex. Our results also indicate that deletions of CIB and ankyrin or mutations that interfere with direct interactions between TMC and CIB proteins abolish the mechanosensory activity of TMCs when they are expressed heterologously in *C. elegans* mechanoinsensitive neurons. Similarly, the function of TMC1/2 channels in mammalian hair cells can also be disrupted or affected by mutations in other genes, such as LHFPL5, TMIE, and CIB2 (Giese et al., 2017, Michel et al., 2017, Wang et al., 2017, Xiong et al., 2012, Zhao et al., 2014). Therefore, TMC channels may not be intrinsically mechanosensitive but require CIB and ankyrin tether proteins to accomplish their mechanosensitivity, as the case for the MEC-4 DEG/ENaC channel of *C. elegans* touch receptor neurons (Goodman et al., 2002). However, a recent study shows that the N-terminal and C-terminal truncated CmTMC1 and MuTMC2 channels can be activated by mechanical force in reconstituted proteoliposomes (Jia et al., 2020). Given that both extracellular (tip links) and intracellular (CIB-ankyrin) tethers bind to the TMC N termini, it is possible that the mechanosensitivity of TMC channels can be tuned exquisitely by tethers to detect small-scale mechanical stimuli relevant for hearing and gentle touch. Moreover, our observation of heterologous TMC-dependent mechanical responses in the ASK cell body (paralleling previous results [Wang et al., 2016] on TMC-dependent chemical responses in ASI neurons) suggests additional proteins may be required to localize TMC sensory complexes to dendritic endings.

### The Gating Spring of TMC Mechanotransduction Channels

The transduction channels in vertebrate hair cells are thought to be mechanically gated by an elastic element called the “gating spring.” The molecular identity of the gating spring has been an open question for decades. The filamentous tip link, consisting of PCDH15 and CDH23, has been put forward as the best candidate to date (Assad et al., 1991, Bartsch et al., 2019). However, some evidence suggests that intracellular elastic tethers might be better candidates for the gating spring. First, transduction channel proteins need to be anchored to the cytoskeleton to resist forces generated by the hair-bundle deflection, because only ∼10–20 pN is enough to extract an unanchored transmembrane protein from the plasma membrane (Bell, 1978). Second, the ultrastructure of tip links suggests that tip links may be too rigid to serve as the gating spring (Kachar et al., 2000). Third, if the tip link were the (only) gating spring, tip link breakage should remove tension in the tip link, thus leading to channel closure. However, there is some evidence suggesting that breaking tip links with BAPTA (1,2-bis(o-aminophenoxy)ethane-N,N,N′,N′-tetraacetic acid) causes the mechanotransduction channel to be open rather than closed (Meyer et al., 1998, Meyer et al., 2005), which contradicts the hypothesis. Consistently, two recent studies show that worm and mouse TMC channels mediate leak currents in *C. elegans* neurons and mouse hair cells (Liu et al., 2019, Yue et al., 2018), suggesting that TMC channels may be continuously open in the absence of tethers. Fourth, previous studies and our results here indicate that *Drosophila*, *C. elegans*, and mammalian TMC proteins are all able to generate mechanosensors in neurons lacking a specialized extracellular tether, such as tip links (Guo et al., 2016, He et al., 2019, Zhang et al., 2016), implying the existence of intracellular tethers to convey force to gate the TMC channel.

Here, we suggest that ankyrin molecules may form the long-sought intracellular gating spring, for several reasons. First, ankyrin, together with *C. elegans* CIB protein CALM-1, co-localizes with TMC-1 in the sensory cilia of the OLQ nose-touch neurons, which are subjected to mechanical forces. Second, ankyrin is specifically required for TMC-mediated mechanosensation in *C. elegans* neurons. Third, the accessory subunit CIB2, which may link ankyrins to TMCs, is essential for mammalian hair cell mechanotransduction. Fourth, atomic force microscopy measurements and molecular dynamics simulations of poly-ankyrin domains indicate that the extensibility and elasticity of large ankyrin-repeat structures well match those predicted by the gating spring model (Lee et al., 2006, Sotomayor et al., 2005). All these results suggest that ankyrin molecules are suitable candidates for the gating spring.

The ability of CIBs to associate with TMCs and ankyrin makes them well suited to serve as intracellular adaptors to physically link the mechanotransduction channel with intracellular tethers. Because both CIB2/3 and PCDH15 directly interact with the TMC1/2 N-terminal domains (Giese et al., 2017, Maeda et al., 2014), it is tempting to speculate that the N-terminal domains of TMC1/2 may convey the force experienced by ankyrins and/or tip links to gate the channel, as shown for the N-terminal ankyrin repeats of *Drosophila* mechanotransduction channel NOMPC (Jin et al., 2017, Zhang et al., 2015b). High-resolution structure of the TMC-CIB-ankyrin channel complex will be valuable for future investigation of how TMC channels are gated by mechanical force.

## STAR★Methods

### Key Resources Table

**Table:** 

REAGENT or RESOURCE	SOURCE	IDENTIFIER
**Antibodies**		

Mouse monoclonal anti-V5	Thermo Fisher Scientific	Cat# R960-25; RRID: AB_2556564
Rabbit polyclonal anti-V5	Abcam	Cat# ab9116; RRID: AB_307024
Rabbit polyclonal anti-KDEL	Thermo Fisher Scientific	Cat# PA1-013; RRID: AB_325593
Rat monoclonal anti-GFP	Chromotek	Cat# 3H9; RRID: AB_10773374
Mouse monoclonal anti-FLAG M2	Sigma-Aldrich	Cat# F3165; RRID: AB_259529
Mouse monoclonal anti-actin	Sigma-Aldrich	Cat# A4700; RRID: AB_476730
Mouse monoclonal anti-V5-HRP	Thermo Fisher Scientific	Cat# R961-25; RRID: AB_2556565
Mouse monoclonal anti-FLAG M2-HRP	Thermo Fisher Scientific	Cat# A8592; RRID: AB_439702
Rabbit polyclonal anti-GFP-HRP	Thermo Fisher Scientific	Cat# A10260; RRID: AB_2534022
Goat anti-Rabbit IgG (H+L) Cross-Adsorbed Secondary Antibody, Alexa Fluor 405	Thermo Fisher Scientific	Cat# A-31556; RRID: AB_221605
Goat anti-Mouse IgG (H+L) Highly Cross-Adsorbed Secondary Antibody, Alexa Fluor 555	Thermo Fisher Scientific	Cat# A-21424; RRID: AB_141780
Goat anti-Mouse IgG (H+L) Highly Cross-Adsorbed Secondary Antibody, Alexa Fluor 568	Thermo Fisher Scientific	Cat# A-11031; RRID: AB_144696
Goat anti-Mouse IgG (H+L) Highly Cross-Adsorbed Secondary Antibody, Alexa Fluor 633	Thermo Fisher Scientific	Cat# A-21052; RRID: AB_2535719
Goat anti-Rabbit IgG (H+L) Highly Cross-Adsorbed Secondary Antibody, Alexa Fluor 488	Thermo Fisher Scientific	Cat# A-11034; RRID: AB_2576217

**Chemicals, Peptides, and Recombinant Proteins**		

Cycloheximide	Sigma-Aldrich	Cat#C4859
cOmplete, EDTA-free Protease Inhibitor Cocktail	Roche	Cat#11873580001
Hygromycin B	Roche	Cat#10843555001
ER-Tracker Green	Thermo Fisher Scientific	Cat#E34251
ER-Tracker Red	Thermo Fisher Scientific	Cat#E34250
Alt-R® S.p. Cas9 Nuclease V3	IDT	Cat#1081058

**Critical Commercial Assays**		

Amersham ECL Prime Western Blotting Detection Reagent	GE Healthcare	RPN2236
QIAshredder	QIAGEN	Cat#79656
Trans-Blot® Turbo Transfer System	Bio-Rad	Cat#1704156
Trans-Blot® Turbo Midi PVDF Transfer Packs	Bio-Rad	Cat#1704157
4–20% Mini-PROTEAN® TGX Stain-Free Protein Gels	Bio-Rad	Cat#4568094
4–20% Criterion TGX Stain-Free Protein Gel	Bio-Rad	Cat#5678094
GFP-Trap Magnetic Agarose	Chromotek	Cat#gtma-20
Anti-v5 agarose affinity gel	Sigma-Aldrich	Cat#A7345
Anti-FLAG® M2 Magnetic Beads	Sigma-Aldrich	Cat#M8823
0.7-mm zirconia beads	BioSpec	Cat#11079107zx
Nunc Lab-Tek chambered coverglass	Thermo Fisher Scientific	Cat#155411PK
Lipofectamine 3000 Transfection Reagent	Thermo Fisher Scientific	Cat#L3000015
Multisite Gateway Three-Fragment cloning system	Thermo Fisher Scientific	Cat#12537-023

**Experimental Models: Cell Lines**		

CHO-K1	ATCC	N/A
HEK293T	ATCC	N/A

**Experimental Models: Organisms/Strains**		

For transgenic arrays, numbers in parentheses indicate DNA injection concentration (ng/μl)		N/A
*tmc-1(ok1859)*	CGC	AQ4537
*calm-1(tm1353)*	CGC	AQ3524
*tmc-1(ok1859); ljEx1266[Pocr-4::tmc-1::SL2mKate2(100); Punc-122::GFP(50)]*	This study	AQ4462
*tmc-1(ok1859); ljEx1267[Pocr-4::tmc-1::SL2mKate2(100); Punc-122::GFP(50)]*	This study	AQ4463
*calm-1(tm1353); ljEx1274[Pocr-4::calm-1::SL2mKate2(100); Punc-122::GFP(50)]*	This study	AQ4470
*calm-1(tm1353); ljEx1275[Pocr-4::calm-1::SL2mKate2(100); Punc-122::GFP(50)]*	This study	AQ4471
*lj120[Loxp::unc-44c::GFP::Loxp]*	This study	AQ4715
*lj120[Loxp::unc-44c::GFP::Loxp]; ljEx1402[Pocr-4::NLS-Cre(50); Punc-122::GFP(50)]*	This study	AQ4745
*ljEx421[Pocr-4::YC3.60]*	This study	AQ2829
*tmc-1(ok1859); ljEx421[Pocr-4::YC3.60]*	This study	AQ4381
*calm-1(tm1353); ljEx421[Pocr-4::YC3.60]*	This study	AQ3639
*tmc-1(ok1859); ljEx421[Pocr-4::YC3.60]; ljEx1266[Pocr-4::tmc-1::SL2mKate2(100); Punc-122::GFP(50)]*	This study	AQ4472
*calm-1(tm1353); ljEx421[Pocr-4::YC3.60]; ljEx1274[Pocr-4::calm-1::SL2mKate2(100); Punc-122::GFP(50)]*	This study	AQ4476
*lj120[Loxp::unc-44c::GFP::Loxp]; ljEx421[Pocr-4::YC3.60]*	This study	AQ4726
*lj120[Loxp::unc-44c::GFP::Loxp]; ljEx421[pocr-4::YC3.60]; ljEx1402[Pocr-4::NLS-Cre(50); Punc-122::GFP(50)]*	This study	AQ4746
*Cas9Is1[tmc-1::GFP;Prps-0::HygR::unc54]*	This study	AQ4230
*calm-1(tm1353); Cas9Is1[tmc-1::GFP;Prps-0::HygR::unc54]*	This study	AQ4232
*Cas9Is1[tmc-1::GFP;Prps-0::HygR::unc54]; ljEx122[Pocr-4:RFP; unc122::GFP]*	This study	AQ4379
*ljIs44[Ptmc-1::GFP]; ljEx940[Psra-9::hCIB2::SL2mKate2(120); Punc-122::RFP(50)]*	This study	AQ3913
*ljEx987[Ptmc-2(3.4kb)::tmc-2 (genomic+3′UTR)::SL2mKate2(50); Punc-122::GFP(50); ljEx543[Psra-9::YC3.60; Punc-122::RFP]*	This study	AQ3914
*ljEx992[Pcalm-1(2.4kb)::calm-1 (genomic)::SL2mKate2(75); Punc-122::GFP(50)]; ljEx543[Psra-9::YC3.60;unc-122::RFP]*	This study	AQ3869
*ljEx992[Pcalm-1(2.4kb)::calm-1 (genomic)::SL2mKate2(75); Punc-122::GFP(50)]; rtIS25[Psra-6::YC2.12, pha-1(+)]*	This study	AQ4163
*ljEx992[Pcalm-1(2.4kb)::calm-1 (genomic)::SL2mKate2(75); Punc-122::GFP(50)]; ljEx421[Pocr-4::YC3.60]*	This study	AQ4384
*ljEx1276[Pocr-4::tmc-1::YFP(100); Punc-122::GFP(50)]*	This study	AQ4477
*calm-1(tm1353); ljEx1276[Pocr-4::tmc-1::YFP(100); Punc-122::GFP(50)]*	This study	AQ4544
*unc-44(e1260); ljEx1276[Pocr-4::tmc-1::YFP(100); Punc-122::GFP(50)]*	This study	AQ4697
*ljEx1276[Pocr-4::tmc-1::YFP(100); Punc-122::GFP(50)]; ljEx1280[Pocr-4::calm-1::mKate2(50); Punc-122::RFP(50)]*	This study	AQ4512
*ljEx1280[Pocr-4::calm-1::mKate2(50); Punc-122::RFP(50)]*	This study	AQ4481
*unc-44(e1260); ljEx1280[Pocr-4::calm-1::mKate2(50); Punc-122::RFP(50)]*	This study	AQ4698
*ljEx1373[Pocr-4::unc-44c::sfGFP(100); Punc-122::GFP(50)]*	This study	AQ4694
*unc-44(e1260); Cas9Is1[tmc-1::GFP;Prps-0::HygR::unc-54 3′UTR]*	This study	AQ4788
*ljEx1170[Pmyo-3::tmc-1::sfGFP(75); Punc-122::RFP(50)]; ljEx1183[Pmyo-3::mCD8::mCherry(50); Punc-122::GFP(50)]*	This study	AQ4242
*ljEx1171[Pmyo-3::tmc-2::sfGFP(75); Punc-122::RFP(50)]; ljEx1183[Pmyo-3::mCD8::mCherry(50); Punc-122::GFP(50)]*	This study	AQ4243
*ljEx1170[Pmyo-3::tmc-1::sfGFP(75); Punc-122::RFP(50)]; ljEx1289[Phim-4::mCherry::atn-1(5); rol-6(50)]*	This study	AQ4520
*ljEx1171[Pmyo-3::tmc-2::sfGFP(75); Punc-122::RFP(50)]; ljEx1289[Phim-4::mCherry::atn-1(5); rol-6(50)]*	This study	AQ4522
*ljEx1170[Pmyo-3::tmc-1::sfGFP(75); Punc-122::RFP(50)]; ljEx1288[Pmyo-3::calm-1::mKate2(2); rol-6(50)]*	This study	AQ4519
*ljEx1132[Pmyo-3::calm-1::mKate2(10); Punc-122::GFP(50)]; WB141{pat-6(st561)IV; zpEx99[pat-6::GFP+rol-6(su1006)]}*	This study	AQ4460
*ljIS131[pmyo-3::GCaMP3-SL2-tagRFP-T]IV*	This study	AQ2953
*tmc-1(ok1859); ljIS131[Pmyo-3::GCaMP3-SL2-tagRFP-T]IV*	This study	AQ4156
*tmc-2(ok1302); ljIS131[Pmyo-3::GCaMP3-SL2-tagRFP-T]IV*	This study	AQ4513
*tmc-1(ok1859); tmc-2(ok1302); ljIS131[Pmyo-3::GCaMP3-SL2-tagRFP-T]IV*	This study	AQ4157
*tmc-1(ok1859); ljIS131[Pmyo-3::GCaMP3-SL2-tagRFP-T]IV; ljEx1291[Pmyo-3::tmc-1::SL2mKate2(80); Pmyo-2::mCherry(5)]*	This study	AQ4516
*tmc-1(ok1859); ljIS131[Pmyo-3::GCaMP3-SL2-tagRFP-T]IV; ljEx1292[Pmyo-3::hTMC1::SL2mKate2(80); Pmyo-2::mCherry(5)]*	This study	AQ4517
*calm-1(tm1353); ljIS131[Pmyo-3::GCaMP3-SL2-tagRFP-T]IV*	This study	AQ4127
*calm-1(tm1353); ljIS131[Pmyo-3::GCaMP3-SL2-tagRFP-T]IV; ljEx1293[Pmyo-3::calm-1::SL2mKate2(50); Pmyo-2::mCherry(5)]*	This study	AQ4518
*tmc-2(ok1302)*	CGC	AQ2545
*lj104[tmc-1(crispr, 8055-8077 deletion); tmc-2(ok1302)]*	This study	AQ3805
*tmc-1(ok1859); ljEx1156[Pmyo-3::tmc-1::SL2mKate2(50); Punc-122::RFP(50)]*	This study	AQ4160
*tmc-1(ok1859); ljEx1158[Pmyo-3::hTMC1::SL2mKate2(50); Punc-122::RFP(50)]*	This study	AQ4153
*lj104[tmc-1(crispr, 8055-8077 deletion); tmc-2(ok1302)]; ljEx1156[Pmyo-3::tmc-1::SL2mKate2(50); Punc-122::RFP(50)]*	This study	AQ4176
*lj104[tmc-1(crispr, 8055-8077 deletion); tmc-2(ok1302)]; ljEx1157[Pmyo-3::tmc-2::SL2mKate2(50); Punc-122::RFP(50)]*	This study	AQ4152
*lj104[tmc-1(crispr, 8055-8077 deletion); tmc-2(ok1302)]; ljEx1158[Pmyo-3::hTMC1::SL2mKate2(50); Punc-122::RFP(50)]*	This study	AQ4154
*calm-1(tm1353); ljEx992[Pcalm-1(2.4kb)::calm-1 genomic::SL2mKate2(75); Punc-122::GFP(50)]*	This study	AQ3922
*calm-1(tm1353); ljEx1145[Pmyo-3::calm-1::SL2mKate2(50); Punc-122::RFP(50)]*	This study	AQ4130
*lj120[Loxp::unc-44c::GFP::Loxp]; ljEx1404[Prab-3::NLS-Cre(50); Punc-122::RFP(50)]*	This study	AQ4748
*ljEx1174[Pcalm-1(2.4kb)::flag-calm-1-genomic::SL2GFP(50); HygR(50)]*	This study	AQ4193
*ljEx1175[Pcalm-1(2.4kb)::calm-1-genomic-flag::SL2GFP(50); HygR(50)]*	This study	AQ4194
*ljEx1176[Pcalm-1(2.4kb)::calm-1-genomic::SL2-flag-GFP(50); HygR(50)]*	This study	AQ4195
*unc-44(e1260); ljEx421[Pocr-4::YC3.60]*	This study	AQ4699
*ljEX543[Psra-9::YC3.60; Punc-122::GFP]*	This study	AQ3093
*ljEx1077[Psra-9::tmc-1::SL2mKate2(70); Psra-9::YC3.60(100); Punc-122::GFP(50)]*	This study	AQ3985
*ljEx1078[Psra-9::tmc-1::SL2mKate2(70); Psra-9::YC3.60(100); Punc-122::GFP(50)]*	This study	AQ3986
*calm-1(tm1353); ljEx1077[Psra-9::tmc-1::SL2mKate2(70); Psra-9::YC3.60(100); Punc-122::GFP(50)]*	This study	AQ4092
*calm-1(tm1353); ljEx1078[Psra-9::tmc-1::SL2mKate2(70); Psra-9::YC3.60(100); Punc-122::GFP(50)]*	This study	AQ4229
*ljEx1000[Psra-9::ceTMC-2::SL2mKate2(100); Psra-9::YC3.60(125); Punc-122::GFP(30)]*	This study	AQ3831
*calm-1(tm1353); ljEx1000[Psra-9::ceTMC-2::SL2mKate2(100); Psra-9::YC3.60(125); Punc-122::GFP(30)]*	This study	AQ3958
*ljEx944[Psra-9::hTMC1::SL2mKate2(110); Psra-9::YC3.60(125); Punc-122::GFP(30)]*	This study	AQ3753
*ljEx945[Psra-9::hTMC1::SL2mKate2(110); Psra-9::YC3.60(125); Punc-122::GFP(30)]*	This study	AQ3754
*ljEx946[Psra-9::hTMC2::SL2mKate2(110); Psra-9::YC3.60(125); Punc-122::GFP(30)]*	This study	AQ3755
*ljEx947[Psra-9::hTMC2::SL2mKate2(110); Psra-9::YC3.60(125); Punc-122::GFP(30)]*	This study	AQ3756
*lj104[tmc-1 (crispr, 8055-8077 deletion); tmc-2 (ok1302)]; ljEx945[Psra-9::hTMC1::SL2mKate2(110); Psra-9::YC3.60(125); Punc-122::GFP(30)]*	This study	AQ3987
*lj104[tmc-1 (crispr, 8055-8077 deletion); tmc-2 (ok1302)]; ljEx946[Psra-9::hTMC2::SL2mKate2(110); Psra-9::YC3.60(125); Punc-122::GFP(30)]*	This study	AQ3988
*calm-1(tm1353); ljEx945[Psra-9::hTMC1::SL2mKate2(110); Psra-9::YC3.60(125); Punc-122::GFP(30)]*	This study	AQ3760
*calm-1(tm1353); ljEx945[Psra-9::hTMC1::SL2mKate2(110); Psra-9::YC3.60(125); Punc-122::GFP(30)]; ljEx942[Psra-9::hCIB2::SL2mKate2(120); Punc-122::mCherry(50)]*	This study	AQ4538
*calm-1(tm1353); ljEx945[Psra-9::hTMC1::SL2mKate2(110); Psra-9::YC3.60(125); Punc-122::GFP(30)]; ljEx943[Psra-9::hCIB3::SL2mKate2(120); Punc-122::mCherry(50)]*	This study	AQ4539
*calm-1(tm1353); ljEx945[Psra-9::hTMC1::SL2mKate2(110); Psra-9::YC3.60(125); Punc-122::GFP(30)]; ljEx920[Psra-9::calm-1::SL2mKate2(50); Pelt-2::mCherry(50)]*	This study	AQ4540
*calm-1(tm1353); ljEx946[Psra-9::hTMC2::SL2mKate2(110); Psra-9::YC3.60(125); Punc-122::GFP(30)]*	This study	AQ3764
*calm-1(tm1353); ljEx946[Psra-9::hTMC2::SL2mKate2(110); Psra-9::YC3.60(125); Punc-122::GFP(30)]; ljEx942[Psra-9::hCIB2::SL2mKate2(120); Punc-122::mCherry(50)]*	This study	AQ4541
*calm-1(tm1353); ljEx946[Psra-9::hTMC2::SL2mKate2(110); Psra-9::YC3.60(125); Punc-122::GFP(30)]; ljEx943[Psra-9::hCIB3::SL2mKate2(120); Punc-122::mCherry(50)]*	This study	AQ4542
*calm-1(tm1353); ljEx946[Psra-9::hTMC2::SL2mKate2(110); Psra-9::YC3.60(125); Punc-122::GFP(30)]; ljEx920[Psra-9::calm-1::SL2mKate2(50); Pelt-2::mCherry(50)]*	This study	AQ4543
*ljEx1063[Psra-9::Lhfpl5::SL2mKate2(110); Psra-9::YC3.60(125); Punc-122::RFP(50)]*	This study	AQ3945
*ljEx1064[Psra-9::Lhfpl5::SL2mKate2(110); Psra-9::YC3.60(125); Punc-122::RFP(50)]*	This study	AQ3946
*ljEx1065[Psra-9::Tmie::SL2mKate2(110); Psra-9::YC3.60(125); Punc-122::RFP(50)]*	This study	AQ3947
*ljEx1066[Psra-9::Tmie::SL2mKate2(110); Psra-9::YC3.60(125); Punc-122::RFP(50)]*	This study	AQ3948
*ljEX1048[Psra-9::hTMC1-Δ77-86::SL2mKate2(110); Psra-9::YC3.60(125); Punc-122::GFP(50)]*	This study	AQ3899
*ljEX1049[Psra-9::hTMC1-Δ77-86::SL2mKate2(110); Psra-9::YC3.60(125); Punc-122::GFP(50)]*	This study	AQ3900
*ljEX1050[Psra-9::hTMC1-Δ87-96::SL2mKate2(110); Psra-9::YC3.60(125); Punc-122::GFP(50)]*	This study	AQ3901
*ljEX1051[Psra-9::hTMC1-Δ87-96::SL2mKate2(110); Psra-9::YC3.60(125); Punc-122::GFP(50)]*	This study	AQ3902
*ljEX1052[Psra-9::hTMC1-Δ127-136::SL2mKate2(110); Psra-9::YC3.60(125); Punc-122::GFP(50)]*	This study	AQ3903
*ljEX1053[Psra-9::hTMC1-Δ127-136::SL2mKate2(110); Psra-9::YC3.60(125); Punc-122::GFP(50)]*	This study	AQ3904
*lj120[Loxp::unc-44c::GFP::Loxp]; ljEx1078[Psra-9::ceTMC-1::SL2mKate2(70); Psra-9::YC3.60(100); Punc-122::GFP(50)]*	This study	AQ4727
*lj120[Loxp::unc-44c::GFP::Loxp]; ljEx1078[Psra-9::ceTMC-1::SL2mKate2(70); Psra-9::YC3.60(100); Punc-122::GFP(50)]; ljEx1403[Psra-9::NLS-Cre(50); Punc-122::RFP(50)]*	This study	AQ4747

**Oligonucleotides**		

sgRNA targeting sequence: tmc-1: CGCGGTGGTGGTGTGAATAT	This study	N/A
sgRNA targeting sequence: tmc-1: TTGATGAGGATGACTCTCCG	This study	N/A
sgRNA targeting sequence: unc-44: TGTCGAACGAAGGCGATCCA	This study	N/A

### Resource Availability

#### Lead Contact

Further information and requests for resources and reagents should be directed to and will be fulfilled by the Lead Contact, William Schafer (wschafer@mrc-lmb.cam.ac.uk).

#### Materials availability

Materials generated in this study, including strains, plasmids and clones, are freely-available from the Lead Contact upon request.

#### Data and code availability

This study did not generate any unique datasets or code.

### Experimental Model and Subject Details

#### Animals

All *C. elegans* strains were grown at 20°C on NGM plates with OP50. Young hermaphrodite animals were used for all experiments. A complete list of strains used in this study and their genotypes is presented in the Key Resources Table.

#### Microbe strains

The *Escherichia coli* OP50 strain was used as a food source for *C. elegans*.

#### Cell lines

We used either CHO-K1 (derived from female Chinese hamster ovaries) or HEK293T (originally derived from a female human fetus) cells as indicated in the Method Details section. Cells were cultured by standard methods.

### Method Details

#### Plasmids and transgenic strains

For confocal imaging, fluorescence resonance energy transfer (FRET) and biochemistry experiments, cDNAs of human and *C. elegans* TMCs, CIBs and ANKs were subcloned into pEGFP-N1, pcDNA3.1 or pcDNA3.1D/V5-His-TOPO® vectors.

Plasmids for expression in *C. elegans* were constructed using the Multisite Gateway Three-Fragment cloning system (12537-023, Invitrogen).

To generate a transcriptional reporter line for *tmc-2*, a 3.4 kb upstream promoter fragment of the start site of *tmc-2* was PCR amplified from N2 genomic DNA and introduced into pDONR P4-P1R. A 6.8 kb *tmc-2* genomic DNA was also PCR amplified from N2 genomic DNA and cloned into pDONR 221. An SL2mKate2 with let-858 3′UTR in pDONR P2R-P3 was used together with the above constructs to generate the transcriptional fusion construct of *tmc-2*. The reporter construct was injected at 50 ng/μl with 50 ng/μl of coelomocyte GFP co-injection marker (*unc-122p::gfp*). Strains expressing both *tmc-2* and ASK-specific reporter were generated by standard genetic crosses.

To generate a transcriptional reporter line for *calm-1* (F30A10.1), a 2.4 kb upstream promoter fragment of the start site of *calm-1* was PCR amplified from N2 genomic DNA and introduced into pDONR P4-P1R. A 2.2 kb *calm-1* genomic DNA was also PCR amplified from N2 genomic DNA and cloned into pDONR 221. An SL2mKate2 with let-858 3′UTR in pDONR P2R-P3 was used together with the above constructs to generate the transcriptional fusion construct of *calm-1*. The reporter construct was injected at 50 ng/μl with 50 ng/μl of coelomocyte GFP co-injection marker (*unc-122p::gfp*). Strains expressing both *calm-1* and OLQ/ASH/ASK-specific reporters were generated by standard genetic crosses.

Promoters for cell-specific transgenic lines are described as below:

OLQ: *ocr-4* (4.8 kb); ASH: *sra-6* (3 kb); muscle: *myo-3* (2.3 kb); ASK: *sra-9* (3 kb).

#### Generation of CRISPR/Cas9 mediated knock-out and GFP knock-in strains

For CRISPR/Cas9 genome engineering in *C. elegans*, we designed sgRNA sequences using web-based program Optimized CRISPR Design (http://zlab.bio/guide-design-resources), and then expressed sgRNA under the control of the *rpr-1* promoter. To generate out-of-frame indels for *tmc-1* knockout, the sgRNA targets on sequence: CGCGGTGGTGGTGTGAATAT. The plasmid carrying the *C. elegans* codon-optimized *Cas9* was injected at 30 ng/μl together with 100 ng/μl of sgRNA construct and 50 ng/μl of coelomocyte GFP co-injection marker (*unc-122p::gfp*). Two of the generated *tmc-1* indel alleles are the following:

wild-type: …gaatcgcagattcttggaccTAAAGCGTTACCGATATTCACACcaccaccgcggaaatatcca…
*tmc-1(lj104)*: …gaatcgcagattcttggacccaccaccgcggaaatatcca…
wild-type: …tggacctaaagcgttaccgaTAttcacaccaccaccgcggaa…
*tmc-1(lj105)*: …tggacctaaagcgttaccgattcacaccaccaccgcggaa…

To generate GFP knock-in alleles of *tmc-1*, a CRISPR/Cas9-mediated homologous recombination method was used. The sgRNA targets on sequence: TTGATGAGGATGACTCTCCG. The homologous recombination template plasmid was generated by the multisite Gateway system. We used hygromycin B selection to identify homologous recombination events. Expression of the hygromycin-resistance gene was driven by the rps-0 promoter, and a prps-0::HygR::unc-54 3′UTR cassette was inserted to the 3′ end of *GFP::unc-54 3′UTR* in a second position pENTRY vector. Flanking regions containing ∼700 bp of homologous arm from either side of the targeted tmc-1 locus were put into the first and third position pENTRY vector, respectively. The PAM site was mutated before fragments assembly.

We then co-injected 30 ng/μl of the plasmid carrying Ce-Cas9, 100 ng/μl of the tmc-1 sgRNA construct, 30 ng/μl of the homologous recombination template plasmid, 10 ng/μl *hsp-16.41p::peel-1*, 10 ng/μl *rab-3p::mcherry*, 2.5 ng/μl *myo-2p::mcherry* and 5 ng/μl *myo-3p::mcherry*. Injected animals were transferred to new OP50 plates (five animals per plate) and allowed to lay eggs for 2∼3 days at 25°C in the absence of selection. Then hygromycin B was added to the plates to a final concentration of 0.2–0.3 mg/ml. Animals were heat-shocked for 2 h at 34°C to activate Peel-1 toxin driven by heat shock promoter after 2∼3 days of hygromycin B selection. The surviving animals lacked all fluorescent extrachromosomal array markers were singled to fresh hygromycin B+ plates. To confirm that knock-in had occurred and identify homozygous knock-in animals, three primers were used to PCR amplify an 1106 bp fragment for knock-in and an 802 bp fragment for wild type. The primer sequences were:

*tmc-1*-KI-upstream-F: GGAGGAGGAGACAGACTCAGCTCCG
*GFP*-KI-R:CCTGTACATAACCTTCGGGCATGGCAC
*tmc-1*-KI-R: GGAGCAAGTCCACCGGAGGGAGC
The resulting PCR products were sequenced.

#### CRISPR/Cas9 editing of the endogenous unc-44 locus

To generate a strain that would allow us to knock out endogenous ankyrin gene *unc-44* in a cell-specific manner, we introduced two loxP sites into the *unc-44* locus. Briefly, we injected Cas9-sgRNA ribonucleoprotein complexes and synthetic single-stranded oligodeoxynucleotide (ssODN) donor with 35 bp 5′ and 3′ homology arms flanking the insertion to insert one loxP site just upstream of the *unc-44* start codon in an edited strain *ju1413(unc-44::gfp::loxp::3Xflag*) from Yishi Jin’s lab (Chen et al., 2017). In the *ju1413* strain, GFP and one loxP site were inserted after Leu1818 in an exon present in most *unc-44* isoforms. The sgRNA targets on sequence: TGTCGAACGAAGGCGATCCA. The injection mixture was assembled with the following final concentrations:

**Table:** 

Reagent	Volume	Final Concentration
H_2_0	3.7 μl	–
KCl (3M)	1 μl	300 mM
HEPES (1M)	0.2 μl	20 mM
pRF4 (300ng/μl)	1.6 μl	50 ng/μl
ssODN (1μg/μl)	2 μl	200 ng/μl
sgRNA (50 μM)	1 μl	5 μM
Cas9 protein (5 μg/μl)	0.5 μl	0.25 μg/μl
Final Volume	10 μl	

A plasmid containing nuclear localized Cre recombinase (nCre) under cell type specific promoters was injected into the *unc-44* edited strain (AQ4715) by standard gonadal microinjection.

#### Behavioral assays

##### (i) Nose touch assays

For nose touch assays, we prepared fresh assay plates within 3 hours of use by spreading 30 μL of OP50 culture onto nematode growth medium plates. Single worm was placed on the food patch and was given 15 minutes to acclimate. The assay was conducted by allowing each individual animal on the plate to move toward an eyelash that was placed perpendicularly to the path of the worm. For each animal we recorded three different outcomes in response to the mechanical stimulus: a reversal, a head withdrawal and no response. The animals were scored blindly and the experiments were repeated on at least two different days to reduce variation. For statistical analysis we used multiple t tests with false discovery rate at Q = 1%.

##### (ii) Drop test assays

Alkaline pH Avoidance behavior was performed using a dry drop assay on unseeded NGM plates. 20 animals were picked from a culture plate and placed on a plate without food for a few seconds to avoid transferring food to the assay plates. Animals were then transferred to the assay plate and allowed to acclimate for 20 minutes. A capillary was used to delivery the alkaline pH stimulus. In short, a small drop of the alkaline solution is delivered to the path of a forward-moving animal. If the animal stops moving forward and initiates a reversal response or a head withdrawal response, it is scored as a positive response. Each animal was tested five times, and a response rate was calculated for each animal. M9 buffer was used as the vehicle for tests, and the pH of the alkaline solution was adjusted to 12 using NaOH. All solutions were made and used freshly at room temperature to avoid precipitation.

#### Calmcium imaging

##### (i) Calcium imaging of OLQ and ASK neurons

Optical recordings were performed on a Zeiss Axioskop 2 upright compound microscope equipped with a Dual View beam splitter and a Uniblitz Shutter. Individual adult worms (∼24 hours past L4) were glued with Dermabond 2-Octyl Cyanoacrylate glue to pads composed of 2% agarose in extracellular saline (5 mM KCl, 1 mM CaCl_2_, 5 mM MgCl_2_, 20 mM d-glucose and 10 mM HEPES buffer, pH 7.2). For imaging of OLQ neurons, a small amount of worm food OP50 was included in the bath solution. Filter-dichroic pairs for calcium imaging were: excitation, 400–440; excitation dichroic 455; CFP emission, 465–495; emission dichroic 505; YFP emission, 520–550. Images were recorded at 10Hz using an iXon EM camera (Andor Technology) and captured using IQ1.9 software (Andor Technology). Analysis was performed using a custom-written MATLAB (Mathworks) program. A rectangular region of interest (ROI) was drawn surrounding the cell body and for every frame the ROI was shifted according to the new position of the center of mass. Fluorescence intensity, F, was computed as the difference between the sum of pixel intensities and the faintest 10% pixels (background) within the ROI. Fluorescence ratio R = F_Y_/F_C_ of the yellow and red channels after correcting for bleed through was used for computing ratio change, ΔR/R. ΔR/R for calcium traces was equal to (R-R_0_)/ R_0_^∗^100, where R_0_ is the average R within the first 3 s of recording. For statistical quantification ΔR/R was computed as (R_1_-R_0_)/ R_0_^∗^100, where R_0_ and R_1_ are the average R over 0.5 s prior and during the stimulation respectively.

##### (ii) Mechanical stimulation for OLQ and ASK neurons

In order to provide mechanical stimuli for the calcium imaging experiments we used a 50 mm diameter drawn glass capillary with the tip rounded to 10 μm on a flame. We positioned the stimulator using a motorized stage (Polytec/PI M-111.1DG microtranslation stage with C-862 Mercury II controller). For OLQ calcium imaging, the needle was placed perpendicular to the worm’s body at a distance of 150 μm from the side of the nose. For ASK calcium imaging, the needle was placed toward the cell body of ASK because the ectopically expressed TMC proteins was enriched in the cell body. In the ‘‘on’’ phase, the glass tip was moved toward the worm so that it could press (the probe was displaced 8 μm in and out for the duration of the stimulus) or gently vibrate (the probe was displaced 2.5 μm in and out for the duration of the stimulus) against the OLQ cilia in the worm’s nose or the cell body of ASK neurons, and in the ‘‘off ’’ phase the needle was returned to its original position.

##### (iii) Calcium imaging and mechanical stimulation in microfluidics chip

Calcium imaging on day 2 adult animals was performed in a custom-designed microfluidic device capable of applying mechanical stimulation to the worm’s body wall muscles. The devices consist of a worm inlet/outlet, an imaging channel (50 × 50 μm width/height), and three sets of actuated PDMS actuators. It is modified from a previously developed microfluidic devices (Cho et al., 2017). The first and the third set of actuated membranes are to trap individual worms. The second set (300 μm from the first set) is to provide the mechanical stimulation to the worm body. The length (along the AP axis) of the second actuated PDMS membrane is 500 μm.

The imaging was performed on a Leica DMIRB inverted microscope using a 20x air objective (N.A. 0.70) with a Hamamatsu EM-CCD camera (100 ms exposure time, 8 frame rate). Simultaneous dual color imaging was performed using a DV2 beamsplitter (Photometrics) with GFP (em. 520/30 nm) and RFP (em. 630/5005 nm) filter sets. Excitation light for fluorescent imaging was delivered through an LCD projector system. Before calcium imaging, we waited for 2 minutes after loading individual worms to ensure that the activity of body wall muscles reach the baseline before recording the activity. A single 30 psi 1 s mechanical stimulus to the body wall muscles was delivered at t = 10 s after the start of the recording. Videos were recorded for 20 s following stimulus delivery. For analysis of calcium transients, fluorescence intensities for each frame were extracted from selected region of interest using a custom MATLAB script. The GCaMP3/tagRFP ratio (R) between intensity values was computed $\left(R=\frac{I_{G\_ROI}}{I_{R\_ROI}}\right)$ to minimize movement artifacts. GCaMP3 and tagRFP intensities were measured as the mean pixel intensity in selected regions of interest (ROI). Baseline values were computed as the mean R prior to stimulus delivery. Calcium traces were computed as the change in R from the baseline value $\left(\frac{\Delta R}{R_{o}}=\frac{R-R_{o}}{R_{o}}\right)$.

#### NemaFlex

##### (i) Microfluidic device fabrication

The micropillar-based force measurement device, NemaFlex, was fabricated using soft lithography. Master molds used in this study were fabricated using two-layer fabrication process, SU-8 2015 and SU-8 2050 negative photoresist (Microchem) for the first and second layer respectively on a 6” silicon wafer as substrate (University Wafer). First, a 25-μm-tall, circular flat layer was fabricated, which forms the boundary of the arena. On top of this layer, a second layer of 75-μm height was fabricated with cylindrical holes that form the micropillars. This two-layer approach provides a total chamber depth of approximately 100 μm and creates dangling (deformable) pillars of height 75 μm. Each chamber was 3 mm in diameter to house a single animal. 32 chambers were placed in an 8x4 array compatible for 2”x3” glass slide for scoring 32 animals ideally.

Polydimethylsiloxane (PDMS) devices were casted (Sylgard 184 A and B 1:10 by weight; Dow Corning) over the SU-8 mold by curing for ∼2 hours at 75°C. The PDMS replica was then treated in an air-plasma cleaner (Harrick Plasma, Ithaca, NY) for 1 minute and bonded to a 2”x3” glass slide. Bonding was done ensuring the pillars did not collapse or deform. Inlet and outlet holes were cored with a 1mm hole puncher (Accuderm) before bonding. Bonded and cleaned microfluidic pillar arenas were loaded with 5 wt% Pluronic F127 solution through one of the inlet ports and incubated for 30 min. Next, the arena was washed with M9 buffer.

##### (ii) Loading worms into the device

Age synchronized worms were incubated at 20°C for 72 hr on a *E. coli* seeded plate. Single young adult animal was hand-picked and released at the already primed chamber inlet. Animal is then drawn into the chamber by pulling liquid from the outlet using a 1 mL syringe. This reduces the chances of introducing air bubble into the chamber. Residual bacteria were removed from the device by washing the chamber with M9 buffer. Animals are allowed to habituate in the arena for approximately 5 minutes before imaging in the food-free environment.

##### (iii) Image acquisition and image analysis

The imaging was conducted on an inverted microscope (IX70, Olympus, Center Valley, PA) with image acquisition from a CCD camera (Retiga R6, QImaging, Canada). The magnification used was 4X with an image resolution of 1.11 μm/pixel. Images were recorded at 5 frames per second for 40-50 s for each worm (200-250 data points from each animal). All imaging was conducted at a temperature of 20 ± 1°C.

Movies were processed offline using custom routines written in MATLAB (Mathworks, R2014b). Worm diameters were measured manually using software ImageJ 1.48v.

##### (iv) Data analysis

The force value at a cumulative probability of 95% (*f*_*95*_) is taken as the strength of the animal. Two-sample t test was used to determine significance of data from muscle contraction assays.

#### Yeast two-hybrid screening

Yeast two-hybrid screening was performed by Hybrigenics Services, S.A.S., Paris, France (https://www.hybrigenics-services.com). The coding sequence for *Mus musculus* - Tmc1 (amino-acid residues 1-193) (GenBank accession number gi: 119703759) was PCR-amplified and cloned into pB27 as a C-terminal fusion to LexA (N-LexA-Tmc1-C) and into pB66 as a C-terminal fusion to Gal4 DNA-binding domain (N-Gal4-Tmc1-C). The constructs were sequence verified and used as a bait to screen a random-primed mouse inner ear cDNA library constructed into pP6. The prey fragments of the positive clones were amplified by PCR and sequenced at their 5′ and 3′ junctions. The resulting sequences were used to identify the corresponding interacting proteins in the GenBank database (NCBI) using a fully automated procedure.

Yeast two-hybrid mating assays are based on the HIS3 reporter gene (growth assay without histidine). The diploid yeast cells were obtained using a mating protocol with yeast strains transformed with baits and preys constructs. Interaction pairs were tested in duplicate as two independent clones from each diploid were picked for the growth assay. For each interaction, several dilutions (10^−1^, 10^−2^, 10^−3^ and 10^−4^) of the diploid yeast cells (culture normalized at 5 × 10^4^ cells) and expressing both bait and prey constructs were spotted on several selective media. The selective medium lacking tryptophan and leucine was used as a growth control and to verify the presence of both the bait and prey plasmids. The different dilutions were also spotted on a selective medium without tryptophan, leucine and histidine.

#### Confocal microscopy and acceptor photobleaching FRET

CHO-K1 cells seeded on Nunc Lab-Tek chambered coverglass (Thermo Scientific) were maintained in Ham’s F12 Nutrient Mixture (Thermo Fisher Scientific) with 10% fetal bovine serum (Thermo Fisher Scientific) at 37°C under 5% CO_2_ and transfected using Lipofectamine 3000 (Thermo Fisher Scientific) following the manufacturer’s instructions. After 24-48 hours of transfection, cells were washed three times with phosphate buffered saline (PBS) and fixed in 3.7% paraformaldehyde for 15 min at room temperature. Cells were then blocked in blocking buffer (1x PBS, 5% normal serum, 0.3% Triton X-100) for 60 min and probed with primary antibodies. After incubation overnight at 4°C, cells were labeled with Alexa Fluor 405/488/555/568/633 conjugated secondary antibodies (1:1000, Thermo Fisher Scientific). Images were obtained with the Zeiss LSM 710 confocal microscope.

For acceptor photobleaching FRET experiments, transfection and immunostaining of cells were performed as described above. Confocal images were obtained with the Leica TCS SP8 confocal microscope and FRET analysis was performed using the FRET-AB wizard of Leica LAS AF software. FRET was measured from donor (EGFP) de-quenching upon acceptor (Alexa Fluor 555 dye) photobleaching. EGFP and Alexa Fluor 555 dye were respectively excited at 488 nm and 561 nm, respectively. Emission intensities of EGFP before and after photobleaching of Alexa Fluor 555 dye within the region of interest (indicated by white dashed boxes) were compared and used to calculate FRET efficiency with Leica LAS AF analysis software. The distance (R) between donor and acceptor was calculated as R = R_0_ × [(1/E) - 1]^1/6^, where R_0_ (6.3 nm) is the Förster critical distance when the donor is EGFP and the acceptor is Alexa Fluor 555 dye, and E represents FRET efficiency E = (1-F_D_/F_D_’), in which F_D_ and F_D_’ are donor fluorescence intensity before acceptor bleaching and donor fluorescence intensity after acceptor bleaching, respectively.

Live worms were anaesthetized with 25 mM sodium azide before being mounted between 2% agarose pads and microscope coverslips for imaging. Confocal images were taken on a Leica TCS SP8 confocal microscope and the 3-D images were reconstituted with the aid of Volocity software (Perkin Elmer).

#### Cycloheximide Treatment and Western Blotting Assay

For cycloheximide treatment, transiently transfected HEK293T cells were treated with cycloheximide for multiple time periods (as indicated in Figure S2) and analyzed by western blot. For western blot assay, cells were lysed with ice-cold lysis buffer (50 mM Tris-HCl, 150 mM NaCl, 1% Nonidet P-40, 1% SDS, 5 mM EDTA, pH 8.0) supplemented with protease inhibitor cocktails (11873580001, Roche) for 30 min on ice. Cell lysates were then centrifuged at 4°C for 20 min at 14,000 x g through a QIAshredder homogenizer (Cat No./ID: 79656, QIAGEN). This step simultaneously removes insoluble material and reduces the viscosity of the lysates. Supernatants were loaded with SDS-PAGE sample buffer, separated by 4%–20% Mini-PROTEAN® TGX™ gels, and transferred onto PVDF membranes using Trans-Blot® Turbo Transfer System (Bio-Rad). After blocking with the buffer of 5% nonfat dry milk in TBS-T (Tris-buffered saline with 0.05% Tween 20), the membranes were incubated overnight at 4°C with primary antibodies (1:1000 for anti-GFP from Chromotek; 1:4000 for anti-FLAG from Sigma; 1:500 for anti-actin from Sigma), and immunoreactive bands were visualized by using the Amersham ECL Prime Western Blotting Detection Reagent (GE Healthcare).

#### Co-immunoprecipitation and pull down assays

For co-immunoprecipitation (coIP) assays, transfected HEK293T cells were washed three times with ice cold PBS and lysed in lysis buffer (150 mM NaCl, 25 mM Tris-HCl, 1% NP-40, 1 mM EDTA, 5% glycerol and proteinase inhibitor cocktails, pH 7.4) on ice for 30 min with extensively pipetting every 10 min. The insoluble fraction was removed by centrifugation at 16,000 × g for 10 min and the lysates were split into two aliquots, one for immunoblot analysis and the other for coIP. Equal amounts of proteins were immunoprecipitated with 25 μL GFP-Trap magnetic agarose beads (GFP-Trap®_MA, Chromotek) or anti-V5 agarose affinity gel (A7345, Sigma-Aldrich) overnight at 4°C with gentle tumbling. The agarose beads were extensively washed four times with wash buffer (150 mM NaCl, 50 mM Tris-HCl, 0.1% NP-40, 0.5 mM EDTA, pH 7.4). The immunoprecipitated protein complexes were eluted using SDS-PAGE sample loading buffer for 5 min at 95°C. The samples were resolved in SDS-PAGE and transferred to PVDF membranes (Bio-Rad), then subjected to western blot analysis with mouse monoclonal anti-V5-HRP antibody (1:5000, R961-25, Thermo Fisher Scientific), mouse monoclonal anti-FLAG M2-HRP antibody (1:2000, A8592, Sigma-Aldrich) or polyclonal anti-GFP-HRP antibody (1:2000, A10260, Invitrogen).

For pull down assays, GFP- or V5-tagged fusion protein lysate was incubated with GFP-Trap magnetic agarose beads (GFP-Trap®_MA, Chromotek) or anti-V5 agarose affinity gel (A7345, Sigma-Aldrich) for 3 hours at 4°C with gentle tumbling. After extensive washing, GFP- or V5-tagged fusion protein coated beads were further incubated with lysate of HEK293T cells expressing CALM-1-3xFLAG fusion proteins overnight at 4°C. The beads were then extensively washed four times with wash buffer. The pull down protein complexes were eluted using SDS-PAGE sample loading buffer for 5 min at 95°C. The samples were resolved in SDS-PAGE and transferred to PVDF membranes (Bio-Rad), then subjected to western blot analysis.

#### Sequence analysis

The multiple sequence alignment and hydrophobicity/hydrophilicity analysis for TMC proteins, as well as phylogenetic analysis for CIB proteins were all performed in DNAMAN.

#### Immunoprecipitation of FLAG-tagged CALM-1

Transgenic *C. elegans* strains that express *flag::calm-1* (AQ4193) or *calm-1::flag* (AQ4194) or *flag::gfp* (AQ4195, negative control) under the control of the native *calm-1* promoter were used for coIP. A prps-0::HygR::unc-54 3′UTR cassette was used as the co-injection marker for positive selection of all these three strains. Fifteen 10-cm NGM plates of mixed age worms grown with hygromycin B (10843555001, Roche) at 0.25mg/ml were used to get enough protein for mass spectrometry analysis. Specifically, worms were washed off NGM plates, collected by centrifugation and resuspended in lysis buffer (100 mM KCl, 1 mM MgCl_2,_ 50 mM HEPES, 1 mM EGTA, 10% glycerol, 1% NP40, 1% DDM, 0.1% CHS with Protease Inhibitor Cocktail, pH 7.4). Samples were homogenized with 0.7-mm zirconia beads (11079107zx, BioSpec) using a TissueLyser II (QIAGEN) for three times of 1 min at frequency of 30 times/sec, and then centrifuged for 10 minutes at 12,000 g to pellet worm debris. FLAG fusion proteins were pulled down using Anti-FLAG® M2 Magnetic Beads (Sigma-Aldrich), and eluates were analyzed by mass spectrometry.

#### Mass Spectrometry

Bead-bound proteins were prepared for mass spectrometric analysis by in solution enzymatic digestion. Briefly, bead-bound proteins in 40 ul of 50 mM NH4HCO3 were reduced in 10 mM DTT, and then alkylated with 55 mM iodoacetamide. After alkylation, 0.5 ug of Trypsin (Promega, UK) was added and the proteins digested for 1 h at 37°C in a thermomixer (Eppendorf, Germany), shaking at 800 rpm. Following this initial digestion, a further 1 ug of Trypsin (Promega, UK) was added and digestion continued overnight at 37°C. After digestion 1 ul of formic acid was added and the beads centrifuged for 30 s at 14,000 rpm. The supernatant was then removed into a fresh, labeled tube. The resulting peptides were analyzed by nano-scale capillary LC-MS/MS using an Ultimate U3000 HPLC (ThermoScientific Dionex, San Jose, USA) to deliver a flow of approximately 300 nL/min. A C18 Acclaim PepMap100 5 μm, 100 μm x 20 mm nanoViper (ThermoScientific Dionex, San Jose, USA), trapped the peptides prior to separation on a C18 Acclaim PepMap100 3 μm, 75 μm x 250 mm nanoViper (ThermoScientific Dionex, San Jose, USA). Peptides were eluted with a 60 min gradient of acetonitrile (2%v/v to 80%v/v). The analytical column outlet was directly interfaced via a nano-flow electrospry ionisation source, with a hybrid dual pressure linear ion trap mass spectrometer (Orbitrap Velos, ThermoScientific, San Jose, USA). Data dependent analysis was carried out, using a resolution of 30,000 for the full MS spectrum, followed by ten MS/MS spectra in the linear ion trap. MS spectra were collected over an m/z range of 300–2000. MS/MS scans were collected using a threshold energy of 35 for collision induced dissociation. LC-MS/MS data were then searched against a protein database (UniProt KB) using the Mascot search engine program (Matrix Science, UK) [X]. Database search parameters were set with a precursor tolerance of 5 ppm and a fragment ion mass tolerance of 0.8 Da. Two missed enzyme cleavages were allowed and variable modifications for oxidized methionine, carbamidomethyl cysteine, pyroglutamic acid, phosphorylated serine, threonine and tyrosine were included. MS/MS data were validated using the Scaffold program (Proteome Software Inc., USA). All data were additionally interrogated manually.

### Quantification and Statistical Analysis

The number of animals and replicates used per experiment is described in detail in the Methods Details subsection for each assay and in the relevant Figure legends. Statistical analysis was carried out as described in the relevant Figure legends. In general, for nose touch behavioral analysis we used multiple t test with false discovery rate Q = 1%. For FRET and calcium imaging experiments with 3 or more test groups, we used one-way ANOVA with Dunnett’s test. For calcium imaging experiments comparing two groups, we used the unpaired t test.

## Supplemental Information

Document S1. Figures S1–S7 and Tables S1 and S2

Document S2. Article plus Supplemental Information
