# Ankyrin Is An Intracellular Tether for TMC Mechanotransduction Channels

**DOI:** 10.1016/j.neuron.2020.07.031

**Published:** 2020-08-19

**Authors:** Yi-Quan Tang, Sol Ah Lee, Mizanur Rahman, Siva A. Vanapalli, Hang Lu, William R. Schafer

(Neuron *107*, 112–125.e1–e10; July 8, 2020)

It was recently brought to the authors’ attention that the western blots in Figures 2E, 4D, and 4G were labeled in a potentially confusing way. Specifically, the “+” and “−” labels for the first lanes of these western blots were intended to indicate whether these lanes contain the bait (TMC-1 fragment in Figures 2E and 4G and UNC-44 in Figure 4D); the text label indicated the prey in the experiment (CALM-1 in Figures 2E and 4D and UNC-44 in Figure 4G). However, because these text labels were positioned next to the “+” and “−” labels, this might lead to the misunderstanding that there was no prey rather than no bait. In addition, in Figure 4F, the label for UNC-44-V5 in the first lane was inadvertently labeled as “−” instead of the correct “+.” All these labels have been amended, and corrected figures are shown below. While this correction does not affect the findings or the conclusions of the study, the authors would like to apologize for this error and any confusion or inconvenience that the original labeling may have caused.

**Figure 2E:**
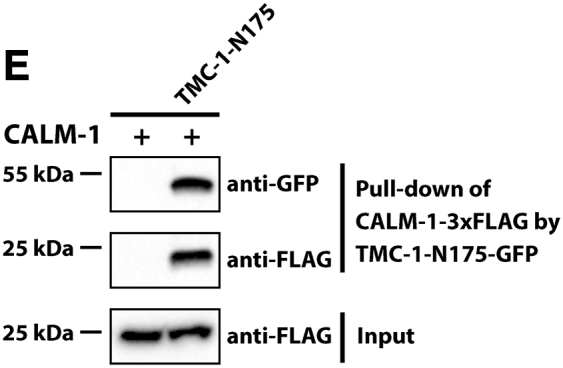
CALM-1 Is Required for TMC-1-Mediated Mechanosensation in OLQ Neurons (corrected)

**Figure 2E:**
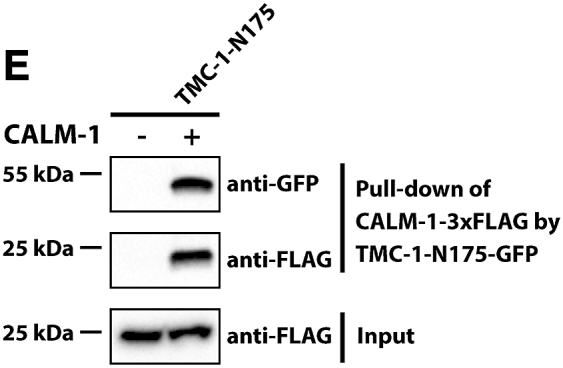
CALM-1 Is Required for TMC-1-Mediated Mechanosensation in OLQ Neurons (original)

**Figure 4:**
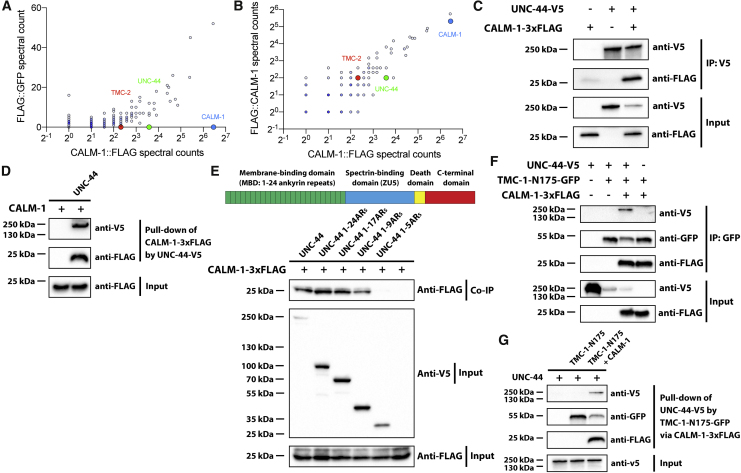
UNC-44/Ankyrin Binds Indirectly to TMC-1 via CALM-1 (corrected)

**Figure 4:**
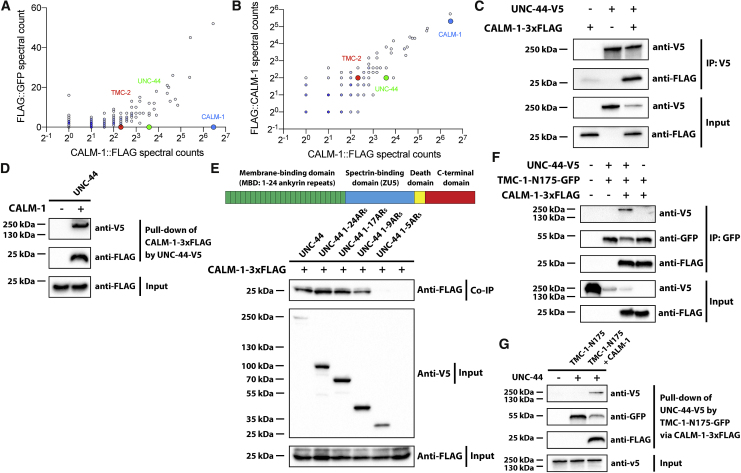
UNC-44/Ankyrin Binds Indirectly to TMC-1 via CALM-1 (original)

