# Supplementary material for: Ankyrin Is An Intracellular Tether for TMC Mechanotransduction Channels
Source: Neuron. 2020 Jul 8;107(1):112–125.e10. doi: 10.1016/j.neuron.2020.03.026 (PMC7343241; doi:10.1016/j.neuron.2020.03.026)
Supplement: Document S1. Figures S1–S7 and Tables S1 and S2 [file mmc1.pdf]

Neuron, Volume *107*

Supplemental Information

Ankyrin Is An Intracellular Tether

for TMC Mechanotransduction Channels

Yi-Quan Tang, Sol Ah Lee, Mizanur Rahman, Siva A. Vanapalli, Hang Lu, and William R. Schafer

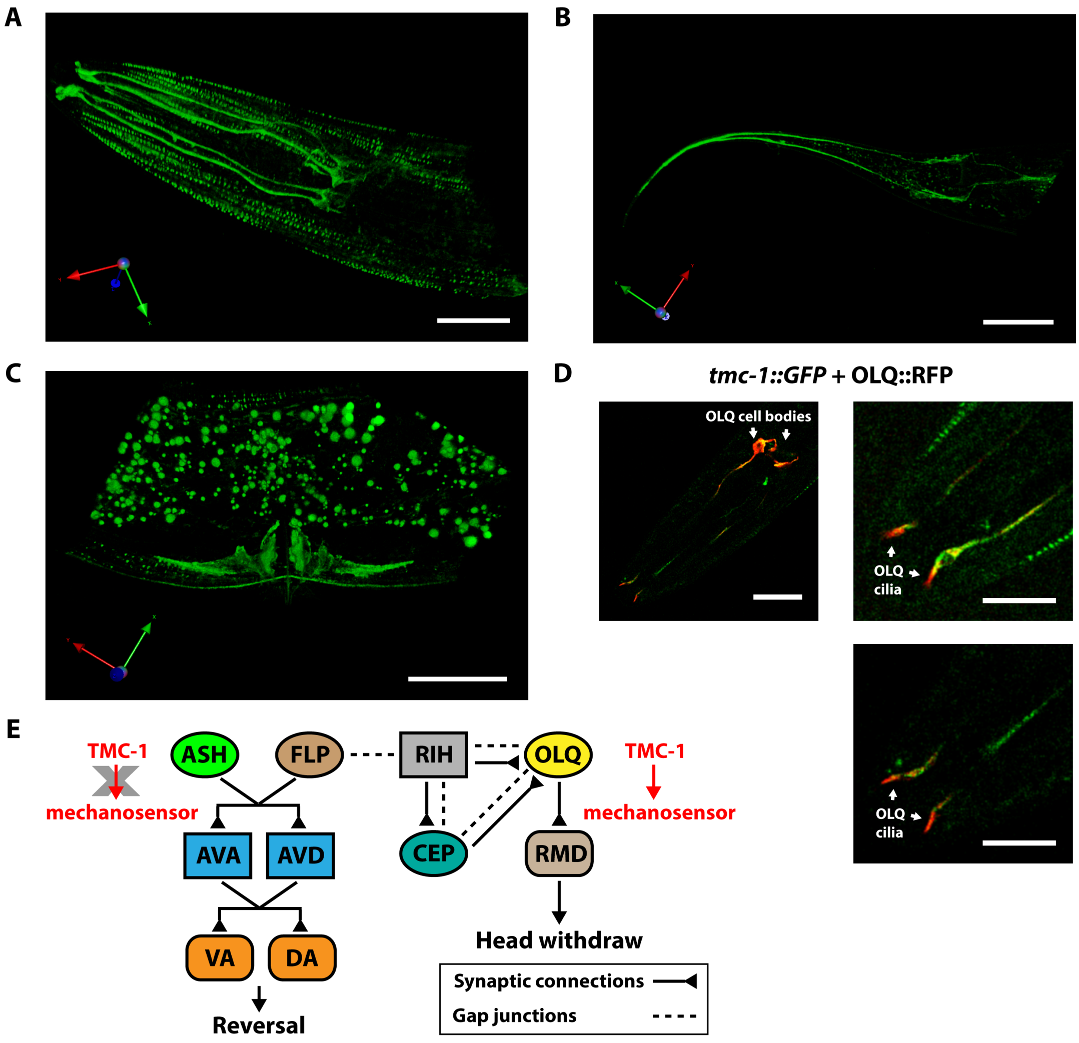

Figure S1. Expression pattern of TMC-1 in *C. elegans*. Related to Figure 1.

(**A**-**C**) The 3D-rendering of endogenous expression of TMC-1::GFP in head neurons, body wall muscles (**A**), tail neurons (**B**), and vulval muscles (**C**). (**D**) Localization of TMC-1::GFP proteins in OLQ cilia of the same animal. *Tmc-1::GFP* knock-in animals express the extrachromosomal array *ljEx122[Pocr-4::RFP]* that specifically labels OLQ neurons with RFP. (**E**) Schematic illustration of the neural circuit for nose-touch behavior. Two- or three-letter designations indicate names of neurons or neural classes; solid or dashed lines indicate synaptic or gap-junction connections. TMC-1 functions in OLQ neurons to promote nose touch-evoked head withdrawal, whereas TMC-1 in ASH neurons does not stimulate reversal in response to nose-touch.

For **A-D**, scale bars represent 10 µm.

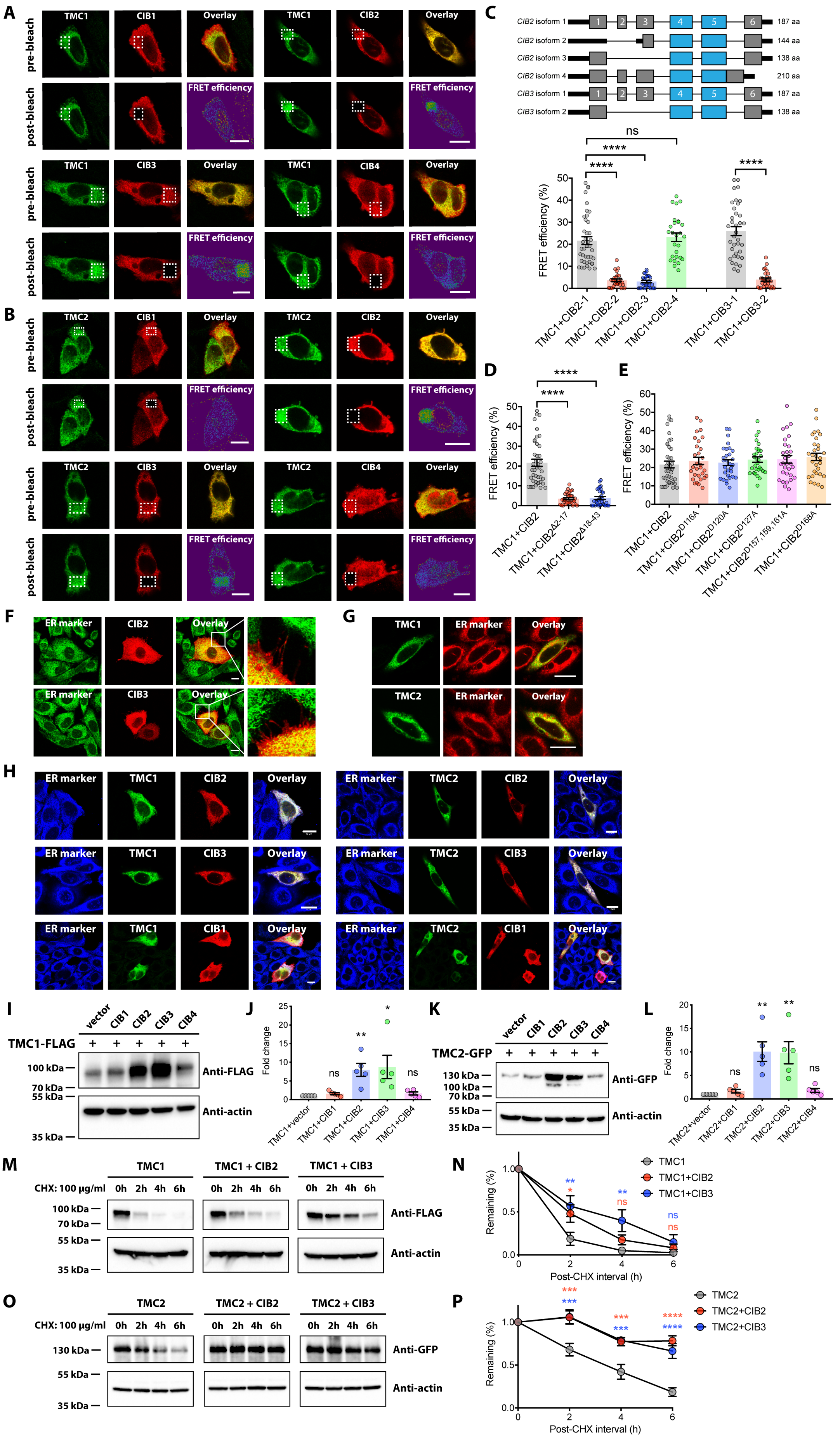

A
B
C
D
E
F
G
H
I
J
K
L
M
N
O
P
pre-bleach
post-bleach
TMC1
TMC2
CIB1
CIB2
CIB3
CIB4
Overlay
FRET efficiency
CIB2 isoform 1
CIB2 isoform 2
CIB2 isoform 3
CIB2 isoform 4
CIB3 isoform 1
CIB3 isoform 2
187 aa
144 aa
138 aa
210 aa
187 aa
138 aa
ns
****
FRET efficiency (%)
TMC1+CIB2-1
TMC1+CIB2-2
TMC1+CIB2-3
TMC1+CIB2-4
TMC1+CIB3-1
TMC1+CIB3-2
TMC1+CIB2
TMC1+CIB2Δ2-17
TMC1+CIB2Δ18-43
TMC1+CIB2D116A
TMC1+CIB2D120A
TMC1+CIB2D127A
TMC1+CIB2D157,159,161A
TMC1+CIB2D168A
ER marker
vector
TMC1-FLAG
100 kDa
70 kDa
55 kDa
35 kDa
Anti-FLAG
Anti-actin
Fold change
TMC1+vector
TMC1+CIB1
TMC1+CIB3
TMC1+CIB4
TMC2-GFP
130 kDa
Anti-GFP
TMC2+vector
TMC2+CIB1
TMC2+CIB2
TMC2+CIB3
TMC2+CIB4
TMC1 + CIB2
TMC1 + CIB3
TMC2 + CIB2
TMC2 + CIB3
CHX: 100 μg/ml
0h
2h
4h
6h
Remaining (%)
Post-CHX interval (h)
TMC1+CIB2
TMC1+CIB3
TMC2+CIB2
TMC2+CIB3

Figure S2. CIB2/3 proteins interact with TMC1/2 to promote their protein stability. Related to Figure 2.

(**A, B**) Acceptor bleaching FRET analysis of CHO-K1 cells transfected with C-terminal EGFP tagged human *TMC1/2* (donor) and C-terminal V5 tagged human *CIB1/2/3/4* (acceptor). Representative confocal images show cells co-expressing *TMC1/2-EGFP* (green) and *CIB1/2/3/4-V5* (red) before and after photobleaching of the acceptor fluorophore (Alexa Fluor 555) within the indicated region (white dashed box). (**C**) FRET analysis of CHO-K1 cells co-expressing human *TMC1* and *CIB2/3* splice isoforms. (Top) Schematics of human *CIB2* and *CIB3* splice isoforms are shown. Sequences encoding EF-hand domains and other coding regions of exons are denoted by blue and gray boxes, respectively. (Bottom) Quantification of FRET efficiency indicates interactions between TMC1 and CIB2-1/CIB2-4/CIB3-1, but not CIB2-2/CIB2-3/CIB3-2. n = 27-41. (**D**) Quantification of FRET efficiency indicates disrupted interactions between TMC1 and N-terminal deletion mutants of CIB2. n = 31-41. (**E**) Quantification of FRET efficiency indicates interactions between TMC1 and indicated EF-hand mutants of CIB2. n = 30-41. (**F**) CIB2/3 proteins localize to the cytoplasm and cell periphery. CHO-K1 cells were transfected with constructs expressing *CIB2/3-V5* fusion proteins and immunostained with anti-V5 and anti-KDEL, a marker for endoplasmic reticulum (ER). (**G**) TMC1/2 proteins were retained in the endoplasmic reticulum (ER) when heterologously expressed in CHO-K1 cells. CHO-K1 cells were transfected with C-terminal EGFP tagged TMC1/2 and stained with ER-Tracker Red dye. (**H**) CIB2/3 proteins were retained in the ER when co-expressed with TMC1/2. CHO-K1 cells were co-transfected with constructs expressing EGFP tagged *TMC1/2* and V5 tagged *CIB1/2/3* and immunostained with anti-V5 and anti-KDEL. (**I-L**) CIB2/3 proteins increase TMC1/2 protein expression. n = 5. (**M-P**) Cycloheximide (CHX, 100 μg/ml) chase experiments show that CIB2/3 proteins increase the half-life of TMC1/2. n = 3.

For **A**, **B**, **F**, **G** and **H**, scale bars represent 10 μm.

For **C**, **D**, **E**, **J**, **L**, **N**, and **P**, error bars indicate SEMs. ns, not significant; * $P < 0.05$; ** $P < 0.01$; *** $P < 0.001$; **** $P < 0.0001$. Statistical analyses were performed using one-way ANOVA with Dunnett's test (**C**, **D**, **E**, **J** and **L**) or two-way ANOVA with Tukey's test (**N** and **P**).

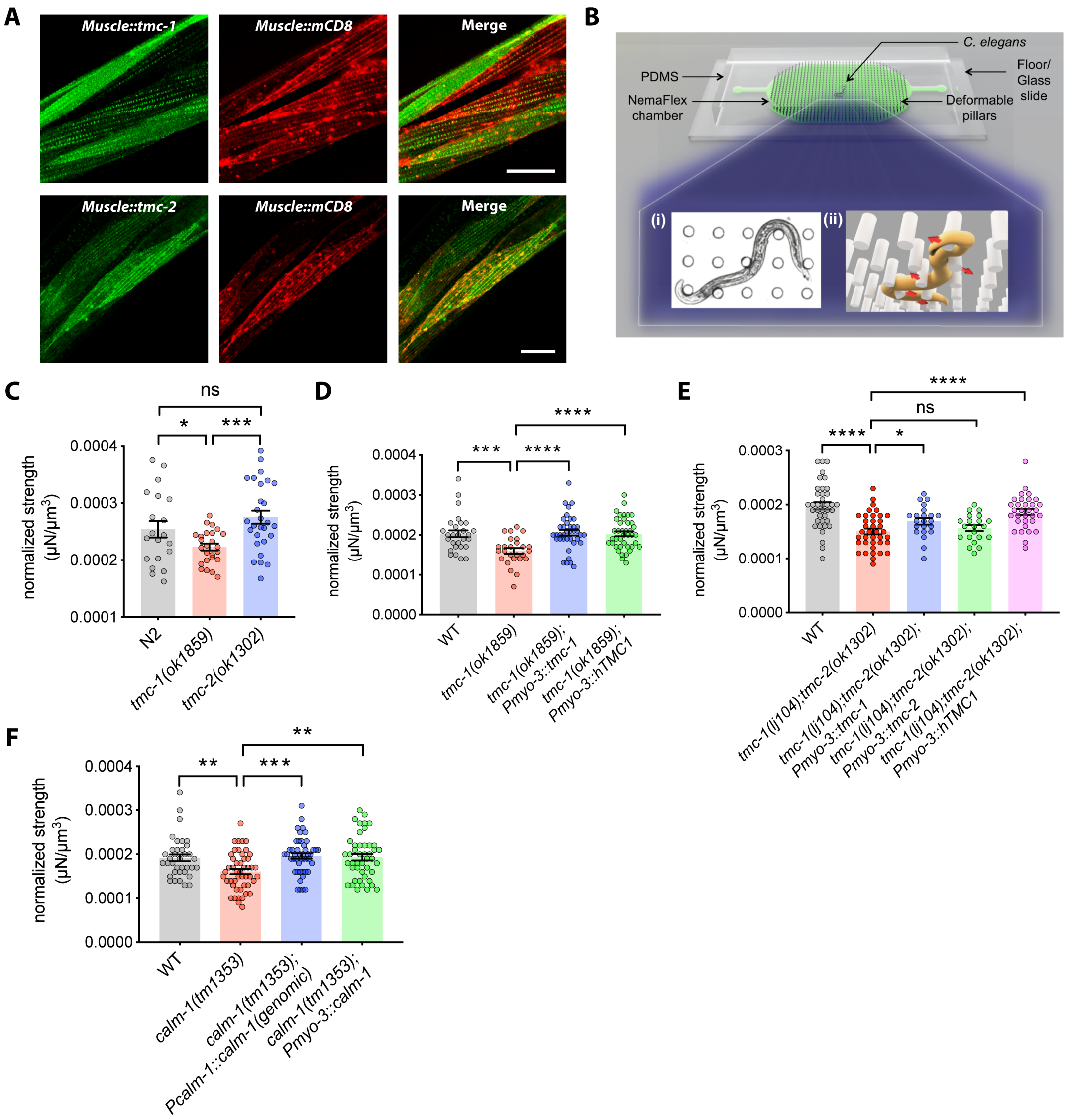

Figure S3. The TMC-1/CALM-1 complex regulates muscular strength in *C. elegans*. Related to Figure 3.

(**A**) Localization of TMC-1::GFP and TMC2::GFP to the plasma membrane as indicated by co-localization with mCD8::mCherry. Scale bars represent 20 µm. (**B**) Schematic of a NemaFlex chamber highlighted as green. NemaFlex chambers are made out of curing transparent PDMS block and bonded irreversibly on a glass slides. Clear glass slide allows imaging of crawling worm and captures deflection of the pillar caused by applied force by the animal. These deflections are then transformed into forces based on the known geometric properties of the device and material properties of PDMS using Timoshenko beam deflection theory. (**B-i**) Bottom view (microscope view) of a crawling worm in micropillar arena. (**B-ii**) Schematic of the

crawling worm to illustrate the deflections. Arrows show the directions of deflection caused by the applied force. (**C**) Quantification of normalized muscular strength for wild-type, *tmc-1(ok1859)* and *tmc-2(ok1302)* mutants. Muscular strength for individual worm was normalized by body diameter, as worm strength is a cubic function of its body diameter. n = 20-28. (**D**) Quantification of normalized muscular strength for wild type, *tmc-1(ok1859)* mutants and rescue lines expressing worm *tmc-1* or human *TMC1* in muscles. n = 26-40. (**E**) Quantification of normalized muscular strength for wild-type, *tmc-1(lj104);tmc-2(ok1302)* mutants and rescue lines expressing worm *tmc-1*, *tmc-2* or human *TMC1* in muscles. n = 21-38. (**F**) Quantification of normalized muscular strength for wild type, *calm-1(tm1353)* mutants and rescue lines expressing *calm-1* genomic sequence using its own promoter or *calm-1* cDNA using a muscle-specific promoter *myo-3p*. n = 36-48.

For **C**, **D**, **E** and **F**, error bars indicate SEMs. ns, not significant; * $P < 0.05$; ** $P < 0.01$; *** $P < 0.001$; **** $P < 0.0001$. Statistical analyses were performed using unpaired t-test.

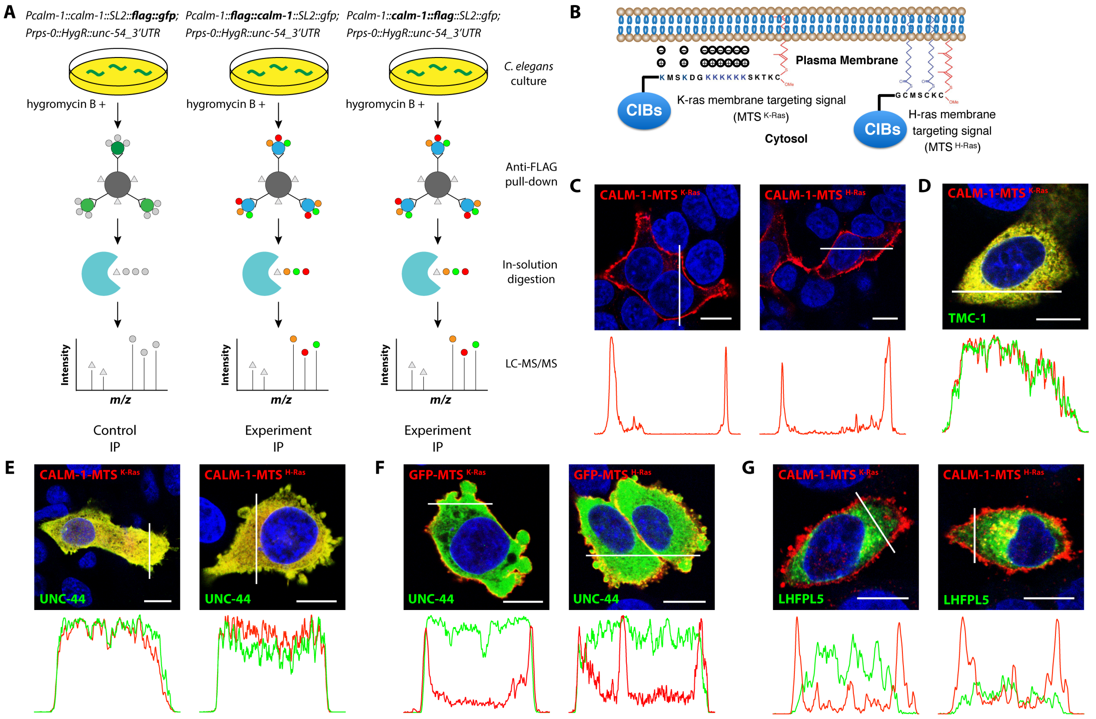

Figure S4. Interactions between ankyrins and CIBs. Related to Figure 4.

(**A**) Schematic diagram outlining the procedure for characterization of CALM-1 interactome using co-immunoprecipitation combined with mass spectrometry. (**B**) Schematic diagram of plasma membrane anchoring of CIBs by addition of membrane targeting signals (MTS), CAAX motifs from K/H-Ras, at their C-termini. (**C**) Representative confocal images for CHO-K1 cells expressing CALM-1-MTS. Intensity histograms for images are shown below the images. (**D, E**) Representative confocal images for CHO-K1 cells expressing CALM-1-MTS with TMC-1 (**D**) or UNC-44 (**E**). Intensity histograms are shown overlapping to facilitate comparison of distribution of two fluorophores. (**F**) Representative confocal images for CHO-K1 cells expressing GFP-MTS and UNC-44. (**G**) Representative confocal images for CHO-K1 cells expressing CALM-1-MTS and LHFPL5.

For **C**, **D**, **E**, **F** and **G**, scale bars represent 10 µm.

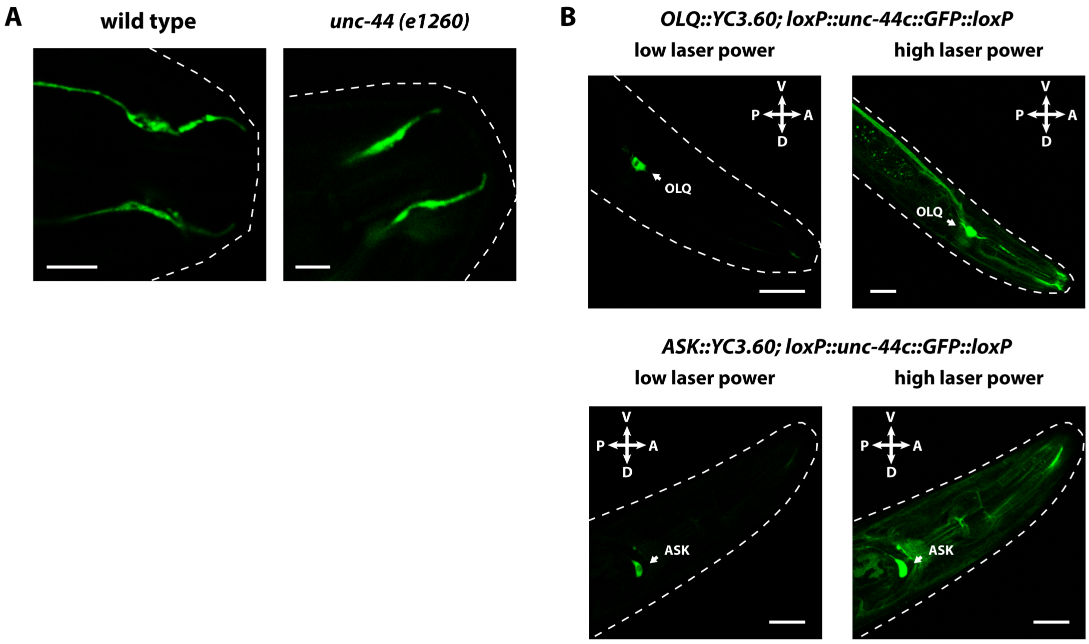

Figure S5. OLQ ciliary structure is retained in *unc-44* mutants. Related to Figure 5.

(**A**) Confocal images of OLQ cilia in wild type animals and *unc-44* mutants show that OLQ ciliary structure is retained in *unc-44* mutants. Scale bars represent 5 µm. (**B**) Confocal images of YC3.60 calcium indicator in OLQ or ASK neurons from *unc-44* edited animals show that CRISPR/Cas9 mediated GFP tagging of endogenous UNC-44 does not interfere OLQ/ASK calcium imaging. Scale bars represent 20 µm.

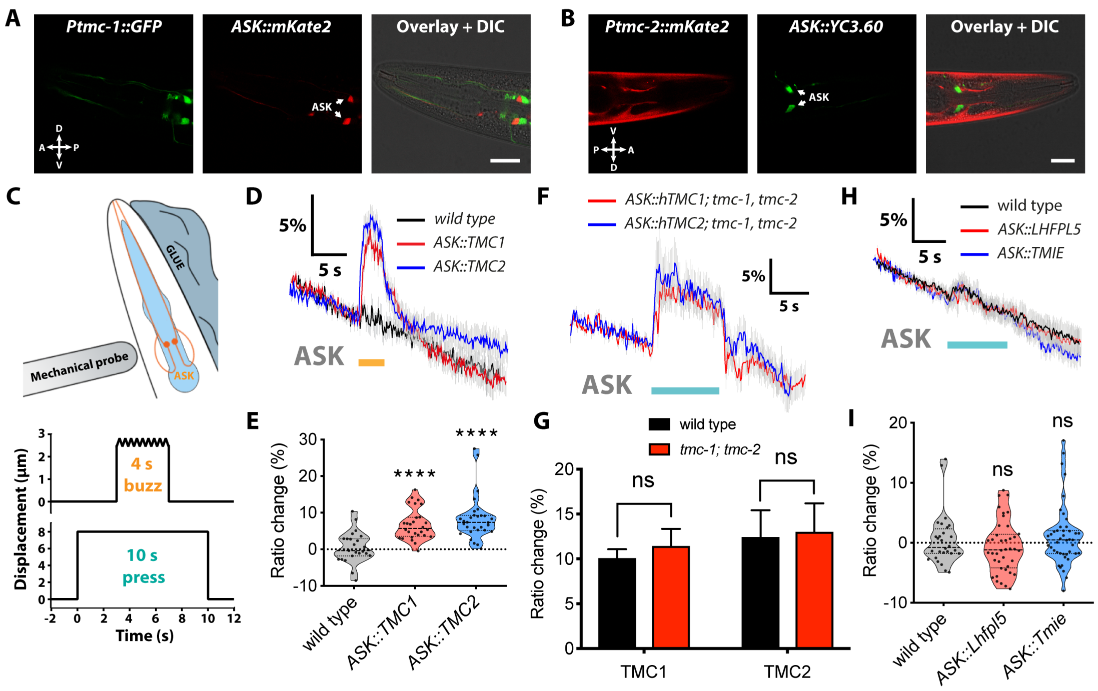

Figure S6. Ectopic expression of TMCs confers mechanosensitivity to ASK neurons. Related to Figure 6.

(A) Lack of expression of *tmc-1* in ASK neurons. Animals express arrays *ljIs44[Ptmc-1::GFP]* and *ljEx940[Psra-9::hCIB2::SL2mKate2]* that specifically labels ASK neurons with *mKate2*. **(B)** Lack of expression of *tmc-2* in ASK neurons. Animals express extrachromosomal arrays *ljEx987[Ptmc-2(3.4kb)::tmc-2 (genomic+3'UTR)::SL2mKate2]* and *ljEx543[Psra-9::YC3.60]* that specifically labels ASK neurons with *YC3.60*. (**C**) Schematic of imaging setup for mechanical stimulation. Worms expressing *YC3.60* in ASK (orange) neurons were immobilized with glue and immersed in a bath solution (top). Two types of mechanical stimulation (press and buzz) were delivered by moving the glass probe against the ASK cell body using a computer-controlled motorized stage (bottom). **(D)** Averaged calcium traces of ASK neurons ectopically expressing human *TMC1/2* in response to 4-s buzz stimulation. (**E**) Quantification of ASK calcium responses for all genotypes in **D**. n = 23-30. (**F**) Averaged calcium traces of ASK neurons ectopically expressing human *TMC1/2* in wild type or *tmc-1*/*tmc-2* double loss-of-function mutants in response to 10-s press stimulation. (**G**) Quantification of ASK calcium responses for each genotype in **F**. n = 22-43. **(H)** Averaged calcium traces of ASK neurons ectopically expressing *Lhfpl5* or *Tmie* in response to 10-s press stimulation. (**I**) Quantification of

ASK calcium responses for all genotypes in **H**. n = 28-43.

For **A** and **B**, scale bars represent 20 μm.

For **D**, **F** and **H**, gray shadings represent SEMs. The duration of the stimulus is shown in cyan or orange.

For **G**, error bars indicate SEMs. For **E** and **I**, data are presented as median with 25th and 75th percentile, and individual data points are plotted as shown. ns, not significant; **** $P < 0.0001$. Statistical analyses were performed using one-way ANOVA with Dunnett's test (**E** and **I**) or unpaired t test (**G**).

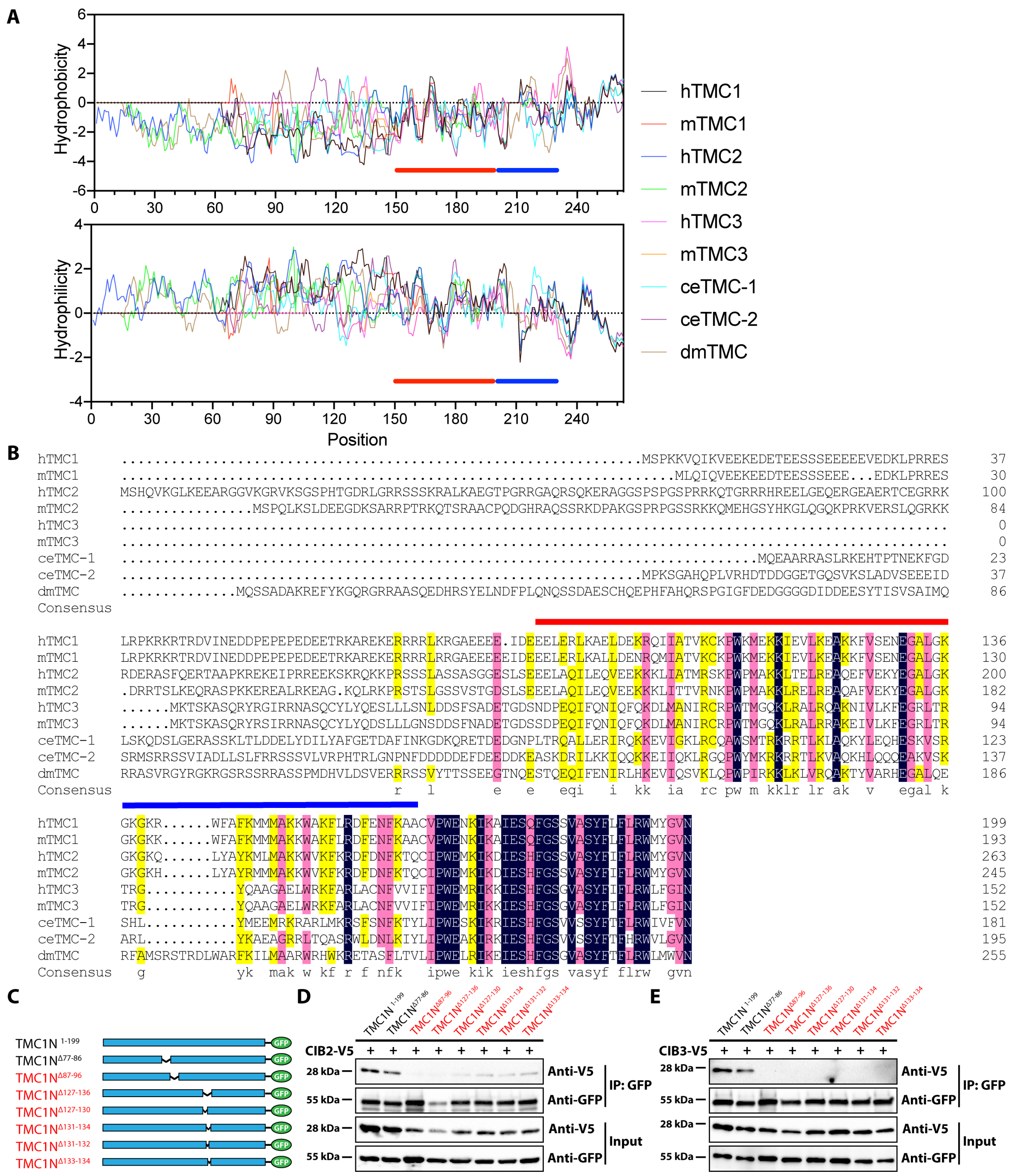

Figure S7. Multiple domains within TMC1 N-terminus are required for the interaction with CIB2/3. Related to Figure 7.

(**A**) Hydrophobicity and hydrophilicity analysis of TMC N-termini. Red and blue lines indicate regions within TMC1 N-terminus required for CIB2 binding as identified in **Figure 7A**. (**B**) Multiple sequence alignment of TMC N-termini. Red and blue lines indicate regions within TMC1 N-terminus required for CIB2 binding as identified in **Figure 7A**. Texts highlighted in dark blue indicate 100% homology level, texts highlighted in pink indicate >=75% homology level, and texts highlighted

in yellow indicate >=50% homology level. (**C**) Schematic representations of N-terminal deletion fragments of TMC1. Thin black lines indicate deleted regions and solid cyan boxes represent cytoplasmic N-terminal coding regions. (**D, E**) Co-immunoprecipitation of CIB2 (**D**) or CIB3 (**E**) with indicated deletion mutants of TMC1 N-terminal regions. Multiple interacting domains within TMC1 N-terminus are required for CIB2/3 binding (marked by red).

Table S1-List of candidates identified in Y2H screening for proteins that interact with TMC1. Related to Figure 2.

Gene ID	Gene name
56506	Cib2
234421	Cib3
12816	Col12a1
192176	Flna
18003	Nedd9
56876	Nsmf

Table S2-List of candidates identified in CALM-1 interactome proteomics. Related to Figure 4.

Identified Proteins	NOTE
CALM-1	an ortholog of human CIB2 (calcium and integrin binding family member 2) and CIB3 (calcium and integrin binding family member 3)
UNC-44	an ortholog of human ANK2 (ankyrin 2) and ANK3 (ankyrin 3)
TMC-2	an ortholog of human TMC1/2/3 (transmembrane channel like 1/2/3)
ACO-2	an ortholog of human ACO2 (aconitase 2)
Y37E3.17	an ortholog of human DMGDH (dimethylglycine dehydrogenase)
UCR-2.2	an ortholog of human UQCRC2 (ubiquinol-cytochrome c reductase core protein 2)
IDHG-2	an ortholog of human IDH3G (isocitrate dehydrogenase (NAD(+)) 3 non-catalytic subunit gamma)
DDX-17	an ortholog of human DDX17 (DEAD-box helicase 17) and DDX5 (DEAD-box helicase 5)
RPS-0	an ortholog of human RPSA (ribosomal protein SA)
HPD-1	an ortholog of human HPD (4-hydroxyphenylpyruvate dioxygenase)
Y69A2AR.18	an ortholog of human ATP5F1C (ATP synthase F1 subunit gamma)
F46G10.1	an ortholog of human KCTD18 (potassium channel tetramerization domain containing 18)
SDHA-1	an ortholog of human SDHA (succinate dehydrogenase complex flavoprotein subunit A)
CCT-8	an ortholog of human CCT8 (chaperonin containing TCP1 subunit 8)
RPS-6	an ortholog of human RPS6 (ribosomal protein S6)
RSP-1	an ortholog of human SRSF6 (serine and arginine rich splicing factor 6)
Y55F3AM.13	no human orthologs
FBXA-64	no human orthologs
